# Synthesis, screening as potential antitumor of new poly heterocyclic compounds based on pyrimidine-2-thiones

**DOI:** 10.1186/s13065-022-00810-4

**Published:** 2022-03-21

**Authors:** El-Sayed M. Abdelrehim, Doaa S. El-Sayed

**Affiliations:** 1 Chemistry Department, Faculty of Science, Damanhour University, Damanhur, Egypt; 2 Chemistry Department, Faculty of Science, Alexandria University, Alexandria, Egypt

**Keywords:** Pyrimidine-2-thiones, [1,2,4]thiadiazolo[4,5-a], Pyrazol-3-ones, [1, 2, 4] triazolo[4,3-a], HCT-116; HepG-2

## Abstract

**Background:**

Continuing our interest in preparing of new heterocyclic compounds and examining their various biological activities, this work was designed to prepare new condensed and non-condensed heterocyclic compounds 9a-c, 10a-c, 11a-c, 13a-c and 14a-c were synthesized starting with pyrimidine-2-thiones 4a-c.

**Results:**

Thiazolo[3,2-a]pyrimidines 9a-c were synthesized by S-alkylation of pyrimidine-2-thiones,4a-c, internal cyclization in alkaline medium with ammonia, condensation with benzaldehyde and finally reaction with hydroxylamine hydrochloride.[1,2,4]thiadiazolo[4,5-a]pyrimidines 11a-c were formed by heating of the 4a-c with benzoylcholride to afford 10a-c followed by reaction with sodium hypochlorite, ammonia and sodium hydroxide. Cyclocondensation of 4a-c with ethyl acetoacetate or formic acid yielded pyrazol-3-ones 13a-c or [1,2,4] triazolo[4,3-a]pyrimidines 14a-c, respectively Elements analysis, IR, 1H-NMR, 13C-NMR and mass spectra were used to validate the structures of newly synthesized heterocycles. Screening of the selected compounds 4a, 6a, 7a, 9a, 10a, 13a and 14a against colon carcinoma cell lines (HCT-116) and hepatocellular carcinoma cell lines (HepG-2).

**Conclusions:**

Elements analysis, IR, 1H-NMR, 13C-NMR and mass spectra were used to validate the structures of newly synthesized heterocycles. Screening of the selected compounds 4a, 6a, 7a, 9a, 10a, 13a and 14a against colon carcinoma cell lines (HCT-116) and hepatocellular carcinoma cell lines (HepG-2) showed that compound 10a exhibited the most cytotoxic, while compounds 4a, 6a and 14a exhibited considerable cytotoxic activity.

**Supplementary Information:**

The online version contains supplementary material available at 10.1186/s13065-022-00810-4.

## Introduction

Continuing our interest in preparing of new heterocyclic compounds and examining their various biological activities [1–6], this work was designed to prepare new derivatives of condensed and non-condensed five-membered rings with pyrimidine. Pyrimidine derivatives have aroused the interest of researchers in recent years, as they have demonstrated a wide variety of biological activities such as antibacterial [6], antiallergic, antihypertensive [7] and antitumor activity [5, 8], along with their cardiopulmonary and bronchodilating effect [9]. It has been observed that the substitution of the benzene ring in pyrimidine derivatives with heterocyclic moieties such as pyrrole and thiophene shows some biological activities such as anti-proliferative and anti-inflammatory activities [10–12]. In addition, the pyrazolpyrimidine and [1, 2,4]triazolopyrimidine derivatives have antimicrobial, antioxidant, antimalarial, analgesic and antitumor activities [13–17]. Most classes of heterocyclic compound have been studied to show their role as strong and chelating ligands with most of transition metals as electron rich sites [1, 18, 19], this point is important in forming novel metal-complexes to be used in different industrial, pharmaceutical and medicinal applications. This study aimed to synthesize and investigate a new heterocyclic class that has an important role in biological behavior based on its structure.

## Results and discussion

### Chemistry

**Scheme 1:**
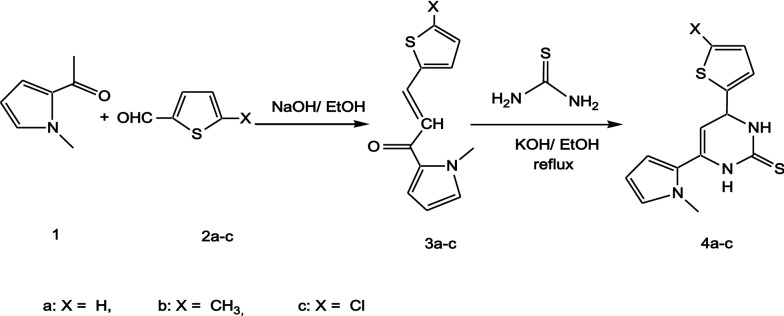
Scheme of preparation of compounds **3a-c** and **4a-c**

The reaction of 2-acetyl-1-methylpyrrole **1** with a series of 5-substituted-thiophene-2-carbaldehyde **2a-c** in alcoholic sodium hydroxide afforded a new series of chalcones **3a-c** as shown in Scheme 1 [20]. Melting points, yield % and IR spectral data of compounds **3a-c** are included in the Additional file 1. The known 3,4-dihydro-1H-pyrimidine-2-thiones **4a-c** nuclei taken as the key synthons for this work were synthesized by cyclocondensation of chalcones **3a-c** with thiourea in the presence of alcoholic potassium hydroxide, Scheme 1 [21]. The structure of compounds **4a-c** was established by their elemental analysis data and their IR spectra which showed two characteristic bands at ύ (3364–3394) and (3215–3275) cm^−1^ for the two NH groups. The ^1^H-NMR spectra of compounds **4a-c** indicated the chemical shifts (δ) at (3.36–3.56) corresponding to the protons of NCH_3_, (4.76–4.94) for H-4 of pyrimidine, (6.08–6.13) for H-5 of pyrimidine, (6.87–7.56) for aromatic protons of pyrrole, (7.51–8.19) for aromatic protons of thiophene and two D_2_O exchangeable singlet peaks at (8.89–10.08) ppm for 2NH groups. The ^13^C-NMR of compound **4a** indicated a group of signals at 39.57 for NCH_3_, 65.37 for C-4 of pyrimidine, 108.30 for C-5 of pyrimidine and a characteristic signal at 176.13 ppm for C=S group, (for more details see the experimental section).

**Scheme 2:**
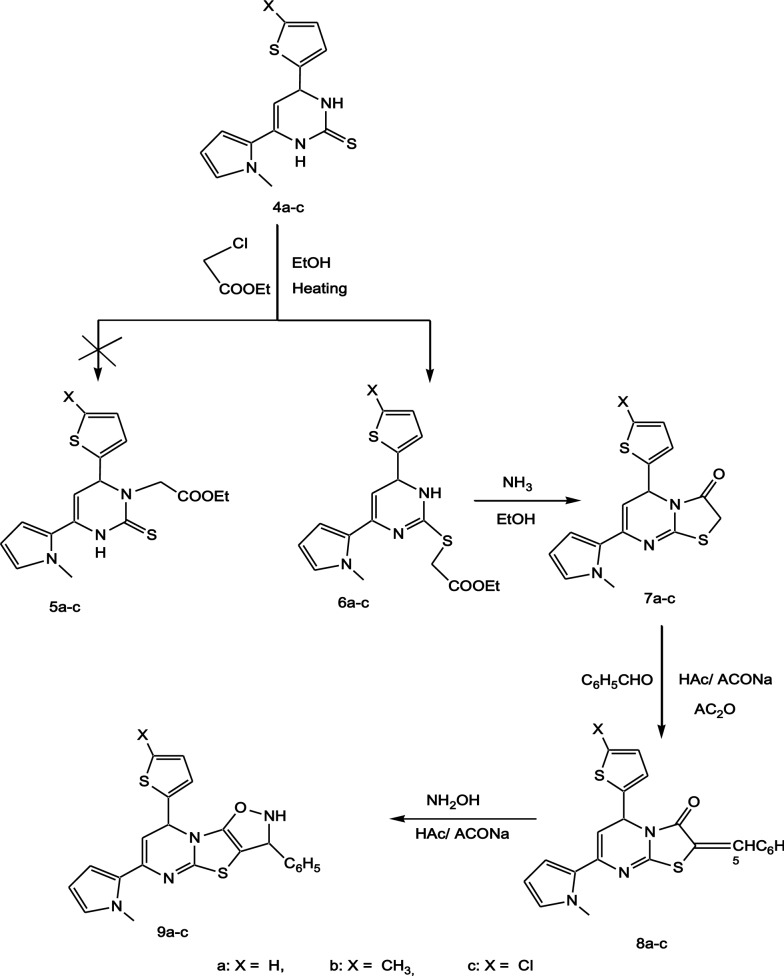
Scheme of preparation of compounds **6a-c**, **7a-c**, **8a-c** and **9a-c**

*S*-alkylation, instead of *N*-alkylation was performed by heating 3,4-dihydro-1H-pyrimidine-2-thione **4a-c** with ethyl chloroacetate to produce ethyl 1,6-dihydro-pyrimidin-2-ylsulfanyl]acetate **6a-c**, Scheme 2 [22]. The structure of the compounds **6a-c** was mainly confirmed from the ^13^C-NMR spectrum of compound **6a** which showed two characteristic signals at 163.74 and 169.87 ppm for C=N and C=O with absence of C=S group signal. The IR spectra of the compounds **6a-c** exhibited stretching bands at (3222–3271) and (1722–1739) cm^−1^ for NH and C=O groups. The ^1^H-NMR of the compounds **6a-c** contained a set of peaks for ethyl, NCH_3_, SCH_2_, pyrimidine, pyrrole, thiophene and NH protons, (for more details see the experimental section). The internal cyclization of dihydropyrimidine esters **6a-c** took place in an alkaline medium using ammonia affording the corresponding thiazolo[3,2-a]pyrimidin-3-ones **7a-c**. The structure of the compounds **7a-c** was confirmed by the disappearance of the NH signals in both the IR and ^1^H-NMR spectra of these compounds, along with the disappearance of ethyl protons in the ^1^H-NMR spectra compared to those in the compounds **6a-c.** In order to build up a fused heterocyclic to the compounds **7a-c**, the compounds **7a-c** were condensed with benzaldehyde in the presence of freshly prepared sodium acetate to give the corresponding 2-Benzylidenethiazolo[3,2-a]pyrimidin-3-ones **8a-c**. Heating under reflux of the compounds **8a-c** with hydroxylamine hydrochloride in the presence of freshly prepared sodium acetate yielded the corresponding isoxazolo[5′,4′:4,5]thiazolo[3,2-a]pyrimidinse **9a-c** [23]. The mass spectrum of 8-(5-Chloro-thiophen-2-yl)-6-(1-methyl-1H-pyrrol-2-yl)-3-phenyl-2,3-dihydro-8H-isoxazolo[5′,4′:4,5]thiazolo[3,2-a]pyr-imidine **9c** has molecular ion peaks at 452 and 454 with in a ratio of 3:1 which is consistent with the molecular formula and the existence of chlorine isotopes of compound **9c**. Also the spectral data of the compounds **9a-c** indicated the presence of NH group at (3207–3233) cm^−1^ and (9.98–10.73) ppm for the IR and ^1^H-NMR spectra, respectively.

**Scheme 3:**
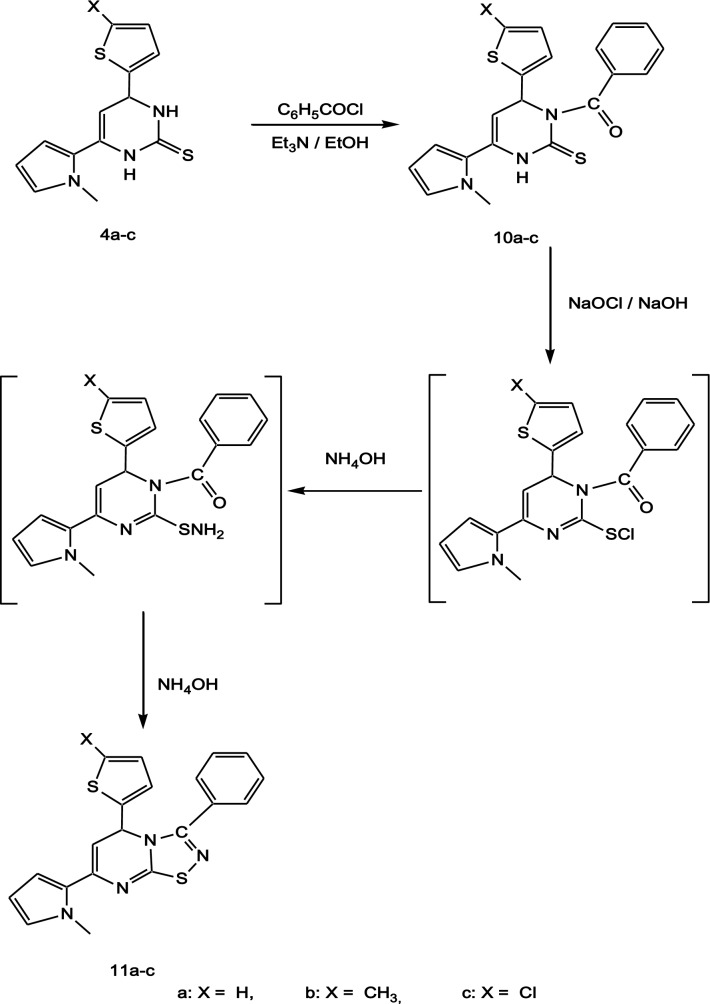
Scheme of preparation of compounds **10a-c** and **11a-c**

The second path way of this work was heating the key synthons **4a-c** under reflux with benzoyl chloride and a few drops of triethylamine to provide compounds **10a-c, **Scheme 3. The structure of the compounds **10a-c** was elucidated by their correct elemental analysis and spectral data, where the IR spectra showed two characteristic bands at (3224–3363) and (1682–1697) cm^−1^ for the NH and C=O groups, respectively. ^1^H-NMR spectra of the compounds **10a-c** showed a characteristic signal at (10.98–11.42) ppm for NH group, along with two characteristic signals at 169.55 and 181.16 ppm for C=O and C=S groups in the ^13^C-NMR spectrum of the compound **10a**. The reaction of the compounds **10a-c** with sodium hypochlorite, ammonia and sodium hydroxide passed through the formation of non-isolable intermediates sulphenyl chloride and sulphenamide which underwent an intramolecular dehydration to produce the corresponding [1, 2, 4]thiadiazolo[4,5-a]pyrimidine** 11a-c,** as shown in Scheme 3 [24]. The elemental analysis of the compounds **11a-c** is consistent with their molecular formula. IR spectra indicated the disappearance of the C=O groups, and the ^13^C-NMR spectrum of the product **11a** also showed the disappearance of the C=S group with two new signals appearing at 150.11 and 156.34 ppm for the two C=N groups which suggesting the formation of thiadiazole ring.

**Scheme 4:**
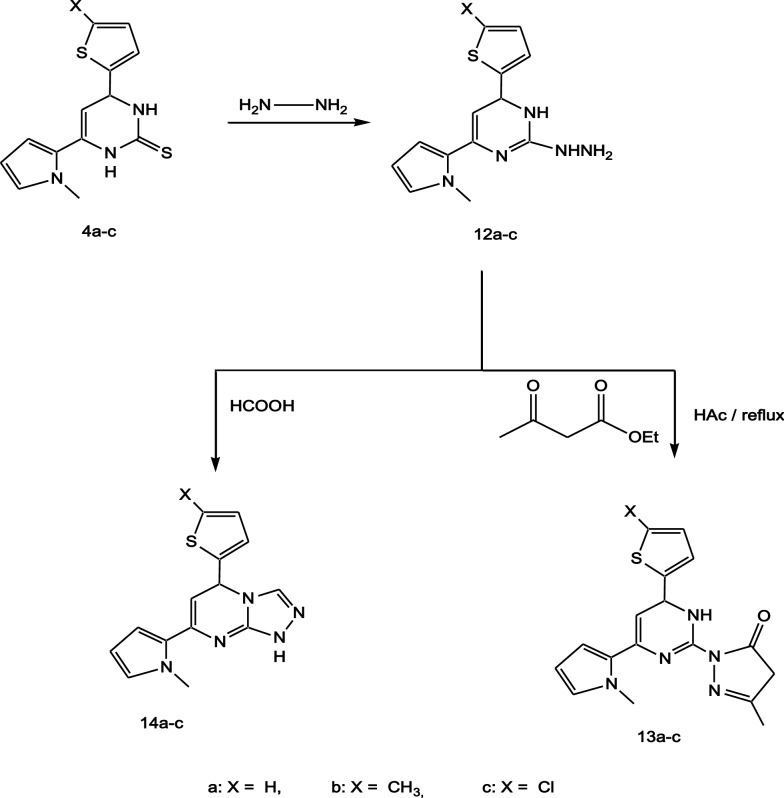
Scheme of preparation of compounds **12a-c**, **13a-c** and **14a-c**

**Fig. 1:**
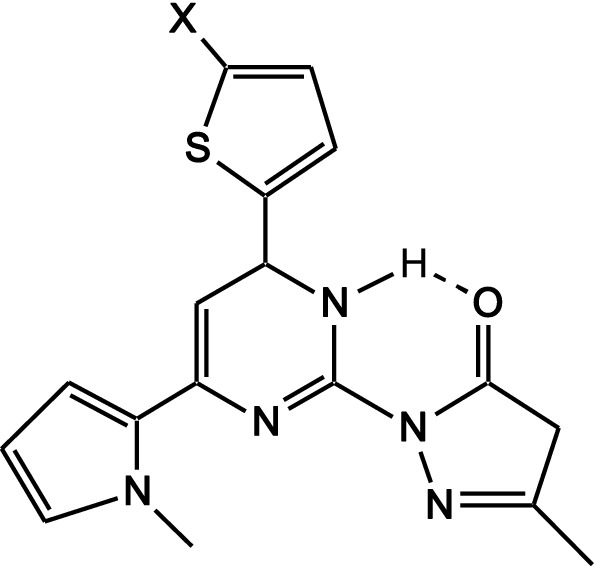
internal H-bonding of compounds **13a-c**

The third part of this work was designed to syntheise the non-condensed system namely: pyrimidopyrazol-3-ones **13a-c** and fused heterocylics compounds triazolo[4,3-a]pyrimidines **14a-c**. Hydrazinolysis of pyrimidine-2-thiones **4a-c** with hydrazine hydrate under reflux gave the corresponding hydrazino compounds **12a-c, **Scheme 4**.** The structures of **12a-c** were established on the basis of their elemental analysis and spectral data, the IR spectra of the compounds **12a-c** showed absorption bands at (3376–3173) cm^−1^ for NH_2_ and NH groups. ^1^H-NMR spectra indicated D_2_O exchangeable singlet signals at (4.46–4.65), (8.79–9.23) and (9.85–10.44) ppm corresponding to the NH_2_, NH of pyrimidine and NH of hydrazine, respectively. Cyclocondensation of the hydrazinopyrimidine compounds **12a-c** with ethyl acetoacetate in acetic acid gave the corresponding [pyrimidin-2-yl]-2,4-dihydro-pyrazol-3-ones **13a-c**. IR spectra of compounds 13a-c revealed absorption bands at (3178–3208) for NH group and at (1683–1691) cm^−1^ for C=O group. The low frequency of the C=O group in the IR spectra for the compounds **13a-c** is due to internal H-bonding as shown in Fig. 1 leads to C=O lengthening and as a result the C=O frequency decreases. ^1^H-NMR spectra of the compounds **13a-c** indicated three singlet signals at (1.75–1.89), (2.73–3.09) and (3.36–3.51) ppm for pyrazolyl CH_3_, pyrazolyl CH_2_ and NCH_3_, respectively along with D_2_O exchangeable singlet signals at (9.38–10.51) ppm corresponding to the NH of pyrimidine. Finally cyclocondensation of **12a-c** with formic acid gave the corresponding [1, 2, 4]triazolo[4,3-a]pyrimidine **14a-c**, the chemical structure of the compounds 14a-c was suggested by their elemental analysis and through the disappearance of the NH and NH_2_ signals in both ^1^H-NMR spectra of **12a-c**.

## Anticancer activity

### Anticancer activity discussion

**Fig. 2:**
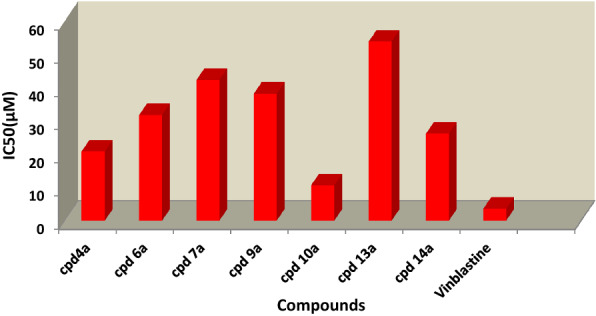
Represent a comparison of IC_50_ (µM) for compounds **4a, 6a, 7a, 9a, 10a, 13a, 14a** and Vinblastine against colon carcinoma cells (HCT-116)

**Fig. 3:**
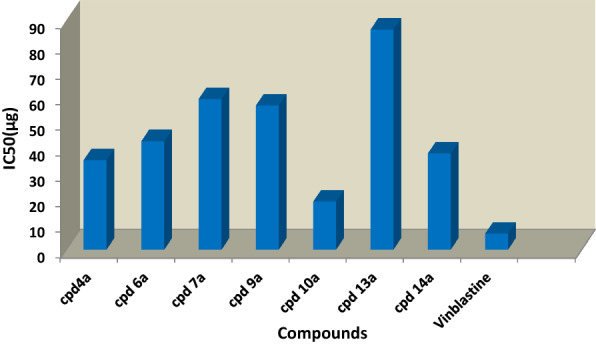
Represent a comparison of IC_50_ (µM) for compounds **4a, 6a, 7a, 9a, 10a, 13a, 14a** and Vinblastine against hepatocellular carcinoma cells lines (HepG-2)

In this study, selected compounds **4a, 6a, 7a, 9a, 10a, 13a** and **14a** were tested for potential cytotoxity using the Mossman [25], Gangadevi and Muthumary [26] methods for anticancer activity against colon carcinoma cells lines (HCT-116) and hepatocellular carcinoma cells lines (HepG-2) using Vinblastine drug as standard. Data on antitumor activity were represented by the cytotoxic effect of the selected compounds. The inhibitory activities of the tested compounds against colon carcinoma cells (HCT-116) and hepatocellular carcinoma cells lines (HepG-2) were calculated by dissolving the selected compounds in DMSO and diluting with saline to appropriate volume using different concentrations of the samples (50, 25, 12.5, 6.25, 3.125 and 1.56 µg mL^−1^), and the cell viability (percent) of the studied compounds was determined using a colorimetric technique, Tables 1, 2. From Tables 1, 2, inhibitory concentration fifty (IC_50_) which corresponds to the concentration necessary for 50% inhabitation of cell viability was calculated, Table 3. Screening of the selected compounds against human colon carcinoma cancer cell lines and hepatocellular carcinoma cells lines revealed that the compound 2-thioxo-3,6-dihydro-2H-pyrimidin-1-yl]-phenyl-methanone **10a** was the most active among the group of selected compounds with IC_50_ (10.72 and 18.95) µM in both human colon carcinoma cancer cell lines and hepatocellular carcinoma cells lines, respectively, Table 3, Figs. 2, 3. Meanwhile, compounds **4a, 6a** and **14a** exhibited considerable cytotoxic action with IC_50_ values ranging from (20.88–31.92) µM in human colon carcinoma cancer cell lines and from (35.22–42.63) µM in hepatocellular carcinoma cells lines. Furthermore, compounds **7a, 9a and 13a** were showed weak cytotoxic action with IC_50_ ranging from (38.32–54.01) µM in human colon carcinoma cancer cell lines and from (56.55–86.33) µM µg in hepatocellular carcinoma cells lines. The data showed that compounds containing a thio group (C=S) such as compounds **4a** and **10a** exhibit the highest cytotoxic activity and this activity increase with the inclusion of a polar group such as the carbonyl group (C=O). The IC_50_ values also show that increased toxicity necessitates larger doses in the case of hepatocellular carcinoma cell lines compared to human colon carcinoma cancer cell lines. As a result, we recommended that the synthesized compound, particularly 2-thioxo-3,6-dihydro-2H-pyrimidin-1-yl]-phenyl-methanone **10a**, be used in the formulation of antibiotics as drugs to increase the sensitivity of antibiotics that stimulate cancer treatment and cause apoptosis in human colon carcinoma.

**Table 1:** Evaluation of cytotoxicity of some chosen synthesized compounds against colon carcinoma cells (HCT-116)

Sample conc. (µg ml^−1^)	Cell viability (%)							
	**4a**	**6a**	**7a**	**9a**	**10a**	**13a**	**14a**	Vinblastine standard
50.000	16.38	26.18	29.78	32.08	14.27	35.18	21.73	12.98
25.000	19.51	37.22	41.05	43.03	17.01	44.91	27.15	16.08
12.500	27.13	46.34	50.68	52.76	23.99	54.45	37.72	20.74
6.250	48.44	70.17	74.29	77.85	44.13	59.90	52.89	39.34
3.125	58.63	85.23	83.55	85.49	52.47	88.11	63.31	48.60
1.560	75.93	91.02	94.47	96.11	66.74	98.22	80.84	59.31
0.000 (DMSO)	100.00	100.00	100.00	100.00	100.00	100.00	100.00	100.00

**Table 2:** Evaluation of cytotoxicity of some chosen synthesized compounds against hepatocellular carcinoma cells lines (HepG-2)

Sample conc. (µg ml^−1^)	Cell viability (%)							
	**4a**	**6a**	**7a**	**9a**	**10a**	**13a**	**14a**	Vinblastine standard
50.000	23.34	30.79	34.92	37.26	19.85	39.61	28.60	15.12
25.000	32.67	40.42	43.43	48.67	24.57	52.16	39.09	17.33
12.500	41.82	52.87	56.33	61.15	35.87	66.03	44.88	24.18
6.250	60.09	69.32	61.38	68.71	52.49	73.52	62.94	46.87
3.125	72.57	84.17	86.89	88.34	61.22	89.56	76.14	55.18
1.560	86.53	94.37	97.09	98.03	78.86	99.12	88.25	73.98
0.000 (DMSO)	100.00	100.00	100.00	100.00	100.00	100.00	100.00	100.00

**Table 3:** IC_50_ (μM) of some chosen synthesized compounds on colon carcinoma cells (HCT-116) and hepatocellular carcinoma cells lines (HepG-2)

Compounds	IC50 (μM)	
	HCT-116	HepG-2
**4a**	20.88 ± 1.88	35.22 ± 2.23
**6a**	31.92 ± 3.67	42.63 ± 4.09
**7a**	42.42 ± 5.54	59.07 ± 6.01
**9a**	38.32 ± 4.01	56.55 ± 5.23
**10a**	10.72 ± 0.83	18.95 ± 1.25
**13a**	54.01 ± 6.09	86.33 ± 7.33
**14a**	26.26 ± 3.09	37.87 ± 2.46
Vinblastine standard	3.61 ± 0.43	6.26 ± 0.69

### In vitro studies

Human colon cancer (HCT-116) cells and hepatocellular carcinoma (HepG-2) cell lines were obtained from the American Type Culture Collection (ATCC, Rockville, MD, USA). The cells were cultured in RPMI-1640 media supplemented with 10% inactivated fetal calf serum and 50 g/mL gentamycin. The cells were kept in a humid environment with 5% CO_2_, 37 °C, and sub-cultured two to three times. Cytotoxic tests on the selected compounds **4a, 6a, 7a, 9a, 10a, 13a** and **14a**: Monolayers of 10,000 cells adhered to the bottom of wells in a 96-well microtiter plate cultured for 24 h at 37 °C in a humidified incubator with 5% CO_2_. The monolayers were then rinsed with sterile phosphate buffered saline (0.01 M pH 7.2), and the cells were incubated at 37 °C with 100 μL of various dilutions of the tested compounds or Vinblastine drug as a control. Six wells were utilized for each concentration of the tested compound, whereas control cells were produced in the absence of the tested compounds. Every 24 h, the observation was performed under an inverted microscope, and the number of (viable) surviving cells was counted by coloring cells with crystal violet, followed by cell lysis with glacial acetic acid (33%), and recording the absorbance at 495 nm, taking into account that the absorption of the untreated cell is 100%.

The following Eq. (1) was used to compute the percentage of cell viability.

1 $${\text{Cell viability \% }} = \left( {1 - { }\frac{ODt}{{ODc}}} \right) \times 100$$

where ODt denotes the mean optical density of test compound-treated wells and ODc denotes the mean optical density of untreated (control) cells. The 50% inhibitory concentration (IC_50_), which is the concentration required to cause toxic effect in 50% of inactivated cells, was estimated from graphic plots.

## Experimental

### Materials and methods

The prepared compounds' melting points are uncorrected and were determined with MEL TEMP II equipment. A Perkin-Elmer FTIR spectrophotometer was used to record the IR spectra (KBr). The NMR spectrum, including ^1^H NMR and ^13^C NMR, was registered on a Bruker spectrometer (400 MHz for ^1^H NMR and 100 MHz for ^13^C NMR) in DMSO-d6 as solvent using tetramethyl-silane (TMS) as internal reference standard. Chemical shift values are expressed in parts per million (ppm) and are abbreviated as follows: (s) for singlet signals, (d) for doublet signals, (t) for triplet signals and (m) for multiplet signals. The NMR spectra were obtained at Kafr Elsheikh University's Faculty of Science. Elements microanalyses were carried out at El-azhr University's Micro Analytical Center. At Cairo University's Micro Analytical Unit, mass spectra were collected using a DI analysis Shimadzu QP-2010 plus mass spectrometer.TLC analytical silica gel plate 60 F254 was used to track the success of the chemical reaction and the purity of the compounds.

### Chemistry

#### General method for synthesis of chalcone 3a-c

3-acetyl-1-methylpyrrole **1** (10 mmol, 1.23 g) was added dropwise to 100 mL of 60% aqueous ethanol solution of sodium hydroxide (30 mmol, 1.20 g) in an ice bath with stirring for 30 min. The 5-substituted thiophene-2-carbaldehyde **2a-c** (10 mmol) was added dropwise over 15 min followed by stirring for 3 h in the ice bath. The reaction mixture was left overnight in a refrigerator, the separated solid was filtered, washed with water, dried and recrystallized from ethanol to give the corresponding 1-(1-Methyl-1H-pyrrol-2-yl)-3-(5-substituted-thiophen-2-yl)-propen- one **3a-c**. Melting points, yield % and IR spectral data of compounds **3a-c** were collected in Table1.

#### General method for synthesis of 6-(1-Methyl-1H-pyrrol-2-yl)-4-(5-substituted-thiophen-2-yl)-3,4-dihydro-1H-pyrimidine-2-thione 4a-c

A mixture of chalcone **3a-c** (10 mmol), thiourea (0.76 g, 10 mmol) and potassium hydroxide (0.85 g, 15 mmol) was heated in 50 mL of absolute ethanol under reflux for 7 h. The reaction mixture was allowed to cool, neutralized with diluted hydrochloric acid, filtrated and washed with water and then the product recrystallized from ethanol to give the corresponding ***4a-c*** compounds.

##### 6-(1-Methyl-1H-pyrrol-2-yl)-4-thiophen-2-yl-3,4-dihydro-1H-pyrimidine-2-thione 4a

According to the previous general method yellow crystals were obtained. Yield (2.23 g, 81%); mp (149–151) °C; IR (KBr) ν_max_: 3377, 32,215 (2 NH), (1612–1589) cm^−1^ (C=C); ^1^H NMR (400 MHz, DMSO-d6) δ 3.42 (S, 3H, NCH_3_), 4.76 (d, 1H, H-4 of pyrimidine, *J* = 7.7 Hz), 6.33- 7.31 (m, 7H, Ar–H protons), 8.89 (s, br, H, 1NH, D_2_O exchangeable), 9.78 ppm (br, 1H, NH, D_2_O exchangeable). ^13^C NMR (100 MHz, DMSO-d6) δ 39.57 (NCH_3_), 65.37 (C-4 of pyrimidine), 108.30 (C-5 of pyrimidine), 119.86, 12.05, 124.58, 127.12,128.66, 130.18 (8 C of aryl carbons), 141.18 (C-6 of pyrimidine), 176.13 ppm (C=S). Anal. Calcd for C_13_H_13_N_3_S_2_ (275.39): C, 56.65; H, 4.72; N, 15.25. Found: C, 56.52; H, 4.65; N, 15.27.

##### 6-(1-Methyl-1H-pyrrol-2-yl)-4-(5-methyl-thiophen-2-yl)-3,4-dihydro-1H-pyrimidine-2-thione 4b

Yellow crystals were obtained according to the previous general method. Yield (2.46 g, 85%); mp (155–157) °C; IR (KBr) ν_max_: 3387, 3263 (2 NH), (1618–1601) cm^−1^ (C=C); ^1^H NMR (400 MHz, DMSO-d6) δ 2.59 (s, 3H, CH_3_), 3.56 (S, 3H, NCH_3_), 4.94 (d, 1H, H-4 of pyrimidine, *J* = 8.0 Hz), 6.08 (d, 1H, H-5 of pyrimidine, *J* = 6.1 Hz), 7.06 (d, 1H, H-3 of pyrrole, *J* = 6.09 Hz), 7.27 (dd, 1H, H-4 of pyrrole), 7.43 (d, 1H, H-5 of pyrrole, *J* = 6.5 Hz), 7.75 (d, 1H, H-3 of thiophene, *J* = 5.0 Hz), 7.92 (d, 1H, H-4 of thiophene, *J* = 5.9 Hz), 9.41 (s, br, H, 1NH, D_2_O exchangeable), 9.96 ppm (br, 1H, NH, D_2_O exchangeable). Anal. Calcd for C_14_H_15_N_3_S_2_ (289.42**):** C, 58.05; H, 5.18; N, 14.51. Found: C, 58.11; H, 5.14; N, 14.43.

##### [6-(5-Chloro-thiophen-2-yl)-4-(1-methyl-1H-pyrrol-2-yl)-3,4-dihydro-1H-pyrimidine-2-thione 4c

Dark yellow crystals were obtained according to the previous general method. Yield (2.70 g, 87%); mp (160–162) °C; IR (KBr) ν_max_: 3394, 3275 (2 NH), (1611–1597) cm^−1^ (C=C); ^1^H NMR (400 MHz, DMSO-d6) δ 3.36 (S, 3H, NCH_3_), 4.83 (d, 1H, H-4 of pyrimidine, *J* = 7.3 Hz), 6.13 (d, 1H, H-5 of pyrimidine, *J* = 6.5 Hz), 6.87 (d, 1H, H-3 of pyrrole, *J* = 5.9 Hz), 7.34 (dd, 1H, H-4 of pyrrole), 7.56 (d, 1H, H-5 of pyrrole, *J* = 6.0 Hz), 7.63 (d, 1H, H-3 of thiophene, *J* = 4.76 Hz), 8.19 (d, 1H, H-4 of thiophene, *J* = 5.6 Hz), 9.65 (s, br, H, 1NH, D_2_O exchangeable), 10.08 ppm (br, 1H, NH, D_2_O exchangeable). Anal. Calcd for C_13_H_12_N_3_S_2_Cl (309.84): C, 50.35; H, 3.87; N, 13.56. Found: C, 50.21; H, 3.90; N, 13.48.

#### General method for synthesis of Ethyl [4-(1-methyl-1H-pyrrol-2-yl)-6-(5-substituted-thiophen-2-yl)-1,6-dihydro-pyrimidin-2-ylsulfanyl]-acetate 6a-c

A solution of 3,4-dihydro-1H-pyrimidine-2-thione ***4a-c*** (5 mmol) and 8 mL of ethyl chloroacetate was heated in 50 mL of absolute ethanol on a water bath for 6 h. The reaction mixture was cooled, neutralized, filtrated and washed with ethyl acetate and then the product was recrystallized from ethanol to give the corresponding ethyl ester ***6a-c***.

##### Ethyl[4-(1-Methyl-1H-pyrrol-2-yl)-6-thiophen-2-yl-1,6-dihydro-pyrimidin-2-ylsulfanyl]acetate 6a

Applying the previous general preparation method pale yellow powder was obtained. Yield (1.34 g, 74%); mp (138–140) °C; IR (KBr) ν_max_: 3222 (NH), 1722 (C=O), 1631(C=N), (1571) cm^−1^ (C=C); ^1^H NMR (400 MHz, DMSO-d6) δ 1.27 (t, 3H, CH_3_ of ethyl group, *J* = 6.9 Hz), 3.48 (S, 3H, NCH_3_), 3.91 (q, 2H,OCH_2_* J* = 7.1 Hz), 4.15 (s, 2H, SCH_2_), 4.93 (d, 1H, H-4 of pyrimidine), 6.27 (d, 1H, H-5 of pyrimidine, *J* = 6.0 Hz), 6.84 (d, 1H, H-3 of pyrrole, *J* = 5.6 Hz), 7.18 (dd, 1H, H-4 of pyrrole), 7.28 (d, 1H, H-5 of pyrrole, *J* = 6.9 Hz), 7.47 (d, 1H, H-3 of thiophene, *J* = 5.18 Hz), 7.73 (dd, 1H, H-4 of thiophene), 8.04 (d, 1H, H-5 of thiophene, *J* = 6.1 Hz), 8.58 ppm (br, 1H, NH, D_2_O exchangeable). ^13^C NMR (100 MHz, DMSO-d6) δ 41.42 (NCH_3_), 46.52 (SCH_2_), 65.17 (C-4 of pyrimidine), 120.09, 123.51, 126.11, 128.69 (4 C of pyrrole) 136.13, 138.21, 139.79, 142.32 (4 C of thiophene), 163.74 (C=N), 169.87 (C=O). Anal. Calcd for C_17_H_19_N_3_O_2_S_2_ (361.48): C, 56.43; H, 5.26; N, 11.62. Found: C, 56.45; H, 5.28; N, 11.57.

##### Ethyl [4-(1-Methyl-1H-pyrrol-2-yl)-6-(5-methyl-thiophen-2-yl)-1,6-dihydro-pyrimidin-2-ylsulfanyl] acetate 6b

Yellow powder was obtained according to the previous general procedure. Yield (1.46 g, 78%); mp (147–149) °C; IR (KBr) ν_max_: 3262 (NH), 1739 (C=O), 1629(C=N), (16,015–1602) cm^−1^ (C=C); ^1^H NMR (400 MHz, DMSO-d6) δ 1.34 (t, 3H, CH_3_ of ethyl group, *J* = 6.5 Hz), 2.46 (s, 3H, CH_3_ of thiophene), 3.58 (S, 3H, NCH_3_), 4.04 (q, 2H,OCH_2_* J* = 7.1 Hz), 4.23 (s, 2H, SCH_2_), 4.99 (d, 1H, H-4 of pyrimidine), 6.61 (s, 1H, H-5 of pyrimidine, *J* = 6.1 Hz), 6.93 (d, 1H, H-3 of pyrrole, *J* = 5.3 Hz), 7.1 (dd, 1H, H-4 of pyrrole), 7.23 (d, 1H, H-5 of pyrrole, *J* = 7.0 Hz), 7.38 (d, 1H, H-3 of thiophene, *J* = 5.3 Hz), 7.89 (d, 1H, H-4 of thiophene*, J* = 6.3 Hz), 8.48 ppm (br, 1H, NH, D_2_O exchangeable). Anal. Calcd for C_18_H_21_N_3_O_2_S_2_ (375.51): C, 57.52; H, 5.60; N, 11.18. Found: C, 57.43; H, 5.55; N, 11.13.

##### Ethyl [6-(5-Chloro-thiophen-2-yl)-4-(1-methyl-1H-pyrrol-2-yl)-1,6-dihydro-pyrimidin-2-ylsulfanyl] acetate 6c

After recrystallization according to the previous general procedure, pale orange powder was obtained. Yield (1.62 g, 82%); mp (157–157) °C; IR (KBr) ν_max_: 3271 (NH), 1732 (C=O), 1624(C=N), (16,011–1603) cm^−1^ (C=C); ^1^H NMR (400 MHz, DMSO-d6) δ 1.23 (t, 3H, CH_3_ of ethyl group, *J* = 6.8 Hz), 3.34 (S, 3H, NCH_3_), 3.89 (q, 2H,OCH_2_* J* = 7.4 Hz), 4.11 (s, 2H, SCH_2_), 4.88 (d, 1H, H-4 of pyrimidine), 6.43 (d, 1H, H-5 of pyrimidine, *J* = 5.78 Hz), 6.81 (d, 1H, H-3 of pyrrole, *J* = 5.3 Hz), 7.2 (dd, 1H, H-4 of pyrrole), 7.11 (d, 1H, H-5 of pyrrole, *J* = 7.5 Hz), 7.23 (d, 1H, H-3 of thiophene, *J* = 5.6 Hz), 7.74 (d, 1H, H-4 of thiophene*, J* = 6.6 Hz), 8.71 ppm (br, 1H, NH, D_2_O exchangeable). Anal. Calcd for C_17_H_18_N_3_O_2_S_2_Cl (395.93): C, 51.52; H, 4.54; N, 10.61. Found: C, 51.47; H, 4.49; N, 10.57.

#### General method for synthesis of 7-(1-Methyl-1H-pyrrol-2-yl)-5-(5-substitued-thiophen-2-yl)-5H-thiazolo[3,2-a]pyrimidin-3-one 7a-c

A solution of ethyl dihydro-pyrimidin-2-yl sulfan- yl]-acetate **6a-c** (5 mmol) in 20 mL of absolute ethanol was treated with ammonia until alkaline (pH > 7) with stirring for 30 min at room temperature. The reaction mixture was allowed to evaporate, the residue was washed with water, dried and recrystallized from n-hexane/ethanol to give thiazolo[3,2-a]pyrimidin-3-one ***7a-c*** at good yields.

##### 7-(1-Methyl-1H-pyrrol-2-yl)-5-thiophen-2-yl-5H-thiazolo[3,2-a]pyrimidin-3-one 7a

According to the previous general method, pale yellow crystals were obtained. Yield (1.32 g, 84%); mp (123–125) °C; IR (KBr) ν_max_: 1701 (C=O), 1632(C=N), 1597 cm^−1^ (C=C); ^1^H NMR (400 MHz, DMSO-d6) δ 3.37 (S, 3H, NCH_3_), 4.15 (s, 2H, SCH_2_), 5.65 (d, 1H, H-4 of pyrimidine), 6.38 (d, 1H, H-5 of pyrimidine, *J* = 6.8 Hz), 6.67 (d, 1H, H-3 of pyrrole, *J* = 6.7 Hz), 7.02 (dd, 1H, H-4 of pyrrole), 7.32 (d, 1H, H-5 of pyrrole, *J* = 6.6 Hz), 7.23 (d, 1H, H-3 of thiophene, *J* = 5.6 Hz), 7.56 (dd, 1H, H-4 of thiophene), 7.89 ppm (d, 1H, H-5 of thiophene, *J* = 6.8 Hz. ^13^C NMR (100 MHz, DMSO-d6) δ 46.11 (NCH_3_), 49.34 (SCH_2_), 67.34 (C-4 of pyrimidine), 121.18, 125.72, 127.32, 129.66 (4 C of pyrrole) 133.45, 135.18, 136.75, 139.12 (4 C of thiophene), 161.15 (C=N), 172.12 ppm (C=O). Anal. Calcd for C_15_H_13_N_3_OS_2_ (315.42): C, 57.07; H, 4.12; N, 13.32. Found: C, 56.98; H, 4.08; N, 13.17.

##### 7-(1-Methyl-1H-pyrrol-2-yl)-5-(5-methyl-thiophen-2-yl)-5H-thiazolo[3,2-a]pyrimidin-3-one 7b

According to the previous general method, yellow crystals were obtained. Yield (1.38 g, 88%); mp (128–130) °C; IR (KBr) ν_max_: 1711 (C=O), 1622(C=N), (1616–1600) cm^−1^ (C=C); ^1^H NMR (400 MHz, DMSO-d6) δ 2.63 (s, 3H, CH_3_ of thiophene), 3.41 (S, 3H, NCH_3_), 4.23 (s, 2H, SCH_2_), 5.63 (d, 1H, H-4 of pyrimidine), 6.45 (d, 1H, H-5 of pyrimidine, *J* = 5.8 Hz), 6.91 (d, 1H, H-3 of pyrrole, *J* = 6.3 Hz), 7.22 (dd, 1H, H-4 of pyrrole), 7.47 (d, 1H, H-5 of pyrrole, *J* = 7.3 Hz), 7.65 (d, 1H, H-3 of thiophene, *J* = 6.9 Hz), 7.97 ppm (d, 1H, H-4 of thiophene, *J* = 7.1 Hz). Anal. Calcd for C_16_H_15_N_3_OS_2_ (329.44): C, 58.28; H, 4.55; N, 12.75. Found: C, 58.17; H, 4.49; N, 12.67.

##### 5-(5-Chloro-thiophen-2-yl)-7-(1-methyl-1H-pyrrol-2-yl)-5H-thiazolo[3,2-a]pyrimidin-3-one 7c

Yellow crystals were obtained According to the previous general method,. Yield (1.49 g, 85%); mp (136–138) °C; IR (KBr) ν_max_: 1702 (C=O), 1627(C=N), (1622–1607) cm^−1^ (C=C); ^1^H NMR (400 MHz, DMSO-d6) δ 3.69 (S, 3H, NCH_3_), 4.47 (s, 2H, SCH_2_), 5.82 (d, 1H, H-4 of pyrimidine), 6.67 (d, 1H, H-5 of pyrimidine, *J* = 6.3 Hz), 6.87 (d, 1H, H-3 of pyrrole, *J* = 6.9 Hz), 7.31 (dd, 1H, H-4 of pyrrole), 7.39 (d, 1H, H-5 of pyrrole, *J* = 6.9 Hz), 7.73 (d, 1H, H-3 of thiophene, *J* = 7.3 Hz), 8.09 ppm (d, 1H, H-4 of thiophene, *J* = 7.6 Hz). (*m/z*): 349 (M^+^, 349, M + 2^+^, 351) (3:1) ratio. Anal. Calcd for C_15_H_12_N_3_OS_2_Cl (349.86): C, 51.45; H, 3.43; N, 12.00. Found: C, 51.39; H, 3.40; N, 11.89.

#### General method for synthesis of 2-Benzylidene-7-(1-methyl-1H-pyrrol-2-yl)-5-(5-substituted-thiophen-2-yl)-5H-thiazolo[3,2-a]pyrimidin-3-one 8a-c

A solution of 5H-thiazolo[3,2-a]pyrimidin-3-one **7a-c** (5 mmol), benzaldehyde (0.53 g, 5 mmol), and freshly prepared sodium acetate (0.41 g, 5 mmol) in 20 mL of glacial acetic acid-acetic anhydride mixture (1:1) was heated under reflux for 5 h. The reaction mixture was left to cool down and poured into ice water, filtered and recrystallized from n-hexane/ethanol to give the corresponding compounds ***7a-c*** at good yields**.**

##### 2-Benzylidene-7-(1-methyl-1H-pyrrol-2-yl)-5-thiophen-2-yl-5H-thiazolo[3,2-a] pyrimidin-3-one 8a

After recrystallization a yellow powder was obtained. Yield (1.51 g, 75%); mp (147–149) °C; IR (KBr) ν_max_: 1694 (C=O), 1628 (C=N), (1622–1607) cm^−1^ (C=C); ^1^H NMR (400 MHz, DMSO-d6) δ 3.49 (S, 3H, NCH_3_), 5.76 (s, 1H, H-4 of pyrimidine, *J* = 8.1 Hz), 6.45 (d, 1H, H-5 of pyrimidine, *J* = 6.2 Hz), (6.42–7.19) (m, 3H of pyrrole), 7.35 (s, 1H, = CH), (7.61–8.23) ppm (m, 8H, of thiophene and phenyl). ^13^C NMR (100 MHz, DMSO-d6) δ 42.65 (NCH_3_), 64.22 (C-4 of pyrimidine), 122.23, 126.15, 128.49, 130.23, 134.47, 136.45, 138.23, 140.55,144.76, 147.88, 149. (18C, C-aryl), (163.10 (C=N), 167.34 ppm (C=O). Anal. Calcd for C_22_H_17_N_3_OS_2_ (403.52): C, 65.42; H, 4.21; N, 10.41. Found: C, 65.33; H, 4.18; N, 10.39.

##### 2-Benzylidene-7-(1-methyl-1H-pyrrol-2-yl)-5-(5-methyl-thiophen-2-yl)-5H-thiazolo[3,2 -a]pyrimidin-3-one 8b

According to the previous general method, dark yellow powder was obtained. Yield (1.52 g, 73%); mp (153–145) °C; IR (KBr) ν_max_: 1700 (C=O), 1623 (C=N), (1615–1601) cm^−1^ (C=C); ^1^H NMR (400 MHz, DMSO-d6) δ 2.47 (s, 3H, CH_3_ of thiophene), 3.36 (S, 3H, NCH_3_), 5.44 (d, 1H, H-4 of pyrimidine, *J* = 7.8 Hz), 6.31 (s, 1H, H-5 of pyrimidine, *J* = 5.9 Hz), (6.56–7.21) (m, 3H of pyrrole), 7.43 (s, 1H, = CH), (7.57–8.17) ppm (m, 7H, of thiophene and phenyl). Anal. Calcd for C_23_H_19_N_3_OS_2_ (417.55): C, 66.10; H, 4.55; N, 10.06. Found: C, 66.02; H, 4.52; N, 10.05.

##### 2-Benzylidene-5-(5-chloro-thiophen-2-yl)-7-(1-methyl-1H-pyrrol-2-yl)-5H-thiazolo[3,2-a]pyrimidin-3-one 8c

According to the previous general method, orange powder was obtained. Yield (1.52 g, 78%); mp (159–161) °C; IR (KBr) ν_max_: 1698 (C=O), 1629 (C=N), (1620–1607) cm^−1^ (C=C); ^1^H NMR (400 MHz, DMSO-d6) δ 3.56 (S, 3H, NCH_3_), 5.79 (d, 1H, H-4 of pyrimidine, *J* = 7.5 Hz), 6.53(s, 1H, H-5 of pyrimidine, *J* = 6.0 Hz), (6.81–7.28) (m, 3H of pyrrole), 7.49 (s, 1H, =CH), (7.65–8.31) ppm (m, 7H, of thiophene and phenyl). (*m/z*): 437 (M^+^, 437, M + 2^+^, 439) (3:1) ratio^.^ Anal. Calcd for C_22_H_16_N_3_OS_2_ Cl (437.97): C, 60.28; H, 3.65; N, 9.59. Found: C, 60.19; H, 3.58; N, 9.53.

#### General method for synthesis of 6-(1-methyl-1H-pyrrol-2-yl)-8-(5-substituted-thiophen-2-yl)-3-phenyl-2,3-dihydro-8H-isoxazolo[5′,4′:4,5]thiazolo[3,2-a]pyrimidine 9a-c

A solution of compound **7a-c** (5 mmol), hydroxylamine hydrochloride (0.35 g, 5 mmol), and freshly prepared sodium acetate (0.41 g, 5 mmol) in 20 mL of glacial acetic acid was heated under reflux for 8 h. The reaction mixture was left to cool down and poured into ice water, filtered and recrystallized from ethyl acetate to give the corresponding compounds ***9a-c*** at good yields**.**

##### 6-(1-methyl-1H-pyrrol-2-yl)-3-phenyl-8-(thiophen-2-yl-2,3-dihydro-8H-isoxazolo[5′,4′:4,5]thiazolo[3,2-a]pyrimidine 9a

After applied the previous procedure a pale yellow powder was obtained. Yield (1.46 g, 70%); mp (172–174) °C; IR (KBr) ν_max_: 3225 (NH), 1617 (C=N), 1595 cm^−1^ (C=C); ^1^H NMR (400 MHz, DMSO-d6) δ 3.38 (S, 3H, NCH_3_), 5.56 (d, 1H, H-4 of pyrimidine), 5.83 (s, 1H, H-3 of isoxazole), 6.68 (d, 1H, H-5 of pyrimidine), (6.63–7.23) (m, 3H of pyrrole), (7.43–8.11) (m, 8H, of thiophene and phenyl), 9.98 ppm (br, 1H, NH, D_2_O exchangeable). ^13^C NMR (100 MHz, DMSO-d6) δ 45.44 (NCH_3_), 64.22 (C-4 of pyrimidine), 74.93 (C-3 of isoxazole), 123.55, 127.67, 128.83, 130.78, 135.40, 137.33, 139.22, 141.43,144.38, 148.49, 149.32 (18C, C-aryl), 161.10 ppm (C=N). Anal. Calcd for C_22_H_18_N_4_OS_2_ (418.54): C, 63.08; H, 4.30; N, 13.38. Found: C, 62.97; H, 4.28; N, 13.34.

##### 6-(1-methyl-1H-pyrrol-2-yl)-8-(5-methyl-thiophen-2-yl)-3-phenyl-2,3-dihydro-8H-isoxazolo[5′,4′:4,5]- thiazolo[3,2-a]pyrimidine 9b

After applied the previous procedure a yellow powder was obtained. Yield (1.58 g, 73%); mp (177–179) °C; IR (KBr) ν_max_: 3207 (NH), 1627 (C=N), (1621–1596) cm^−1^ (C=C); ^1^H NMR (400 MHz, DMSO-d6) δ 2.27 (s, 3H, CH_3_ of thiophene), 3.46 (S, 3H, NCH_3_), 5.41 (d, 1H, H-4 of pyrimidine), 5.65 (s, 1H, H-3 of isoxazole), 6.68 (d, 1H, H-5 of pyrimidine), (6.56–7.29) (m, 3H of pyrrole), (7.28–8.19) (m, 7H, of thiophene and phenyl), 10.54 ppm (br, 1H, NH, D_2_O exchangeable). Anal. Calcd for C_23_H_20_N_4_OS_2_ (432.56): C, 63.81; H, 4.62; N, 12.95. Found: C, 63.85; H, 4.57; N, 12.86.

##### 8-(5-Chloro-thiophen-2-yl)-6-(1-methyl-1H-pyrrol-2-yl)-3-phenyl-2,3-dihydro-8H-isoxazolo[5′,4′:4,5-] thiazolo[3,2-a]pyrimidine 9c

According to the previous general procedure a yellow powder was obtained. Yield (1.72 g, 76%); mp (186–188) °C; IR (KBr) ν_max_: 3233 (NH), 1625 (C=N), (1618–1595) cm^−1^ (C=C); ^1^H NMR (400 MHz, DMSO-d6) δ 3.61 (S, 3H, NCH_3_), 5.69 (d, 1H, H-4 of pyrimidine), 5.91 (s, 1H, H-3 of isoxazole), 6.57 (d, 1H, H-5 of pyrimidine), (6.81–7.33) (m, 3H of pyrrole), (7.42–8.28) (m, 7H, of thiophene and phenyl), 10.73 ppm (br, 1H, NH, D_2_O exchangeable). (*m/z*): 452 (M^+^, 452, M + 2^+^, 454) (3:1) ratio. Anal. Calcd for C_22_H_17_N_4_OS_2_Cl (452.598)**:** C, 58.28; H, 3.75; N, 12.36. Found: C, 58.19; H, 3.72; N, 12.29.

#### General method for synthesis of [4-(1-Methyl-1H-pyrrol-2-yl)-6-(5-substituted-thiophen-2-yl)-2-thioxo-3,6-dihydro-2H-pyrimidin-1-yl]-phenyl-methanone 10a-c

A solution of 3,4-dihydro-1H-pyrimidine-2-thione ***4a-c*** (5 mmol), benzoyl chloride (1.40 g, 10 mmol), and few drops of triethylamine in 25 mL of ethanol was heated under reflux for 4 h. The reaction mixture was left to cool down and poured into ice water, filtered and recrystallized from ethanol to afford compounds ***10a-c*.**

##### [4-(1-Methyl-1H-pyrrol-2-yl)-6-thiophen-2-yl-2-thioxo-3,6-dihydro-2H-pyrimidin-1-yl]-phenylmethan- one 10a

Applying the previous general preparation method yellow powder was obtained. Yield (1.54 g, 81%); mp (161–163) °C; IR (KBr) ν_max_: 3224 (NH), 1682 (C=O), (1605–1547) cm^−1^ (C=C); ^1^H NMR (400 MHz, DMSO-d6) δ 3.38 (S, 3H, NCH_3_), 5.51 (d, 1H, H-4 of pyrimidine, *J* = 6.7 Hz), 6.19 (d, 1H, H-5 of pyrimidine, *J* = 7.6 Hz), 6.65 (d, 1H, H-3 of pyrrole, *J* = 6.8 Hz), 7.07 (dd, 1H, H-4 of pyrrole), 7.22 (d, 1H, H-5 of pyrrole, *J* = 6.4 Hz), (7.47–8.35) (m, 8H, of thiophene and phenyl), 10.98 ppm (br, 1H, NH, D_2_O exchangeable). ^13^C NMR (100 MHz, DMSO-d6) δ 49.23 (NCH_3_), 61.96 (C-4 of pyrimidine), 121.78, 125.63, 128.01, 130.12, 136.63, 138.45, 140.33 145.08, 147.24 (16C, C-aryl), 169.55 (C=O), 181.16 ppm (C = S). Anal. Calcd for C_20_H_17_N_3_OS_2_ (379.50): C, 63.24; H, 4.48; N, 11.07. Found: C, 63.18; H, 4.42; N, 10.97.

##### [4-(1-Methyl-1H-pyrrol-2-yl)-6-(5-methyl-thiophen-2-yl)-2-thioxo-3,6-dihydro-2H-pyrimidin-1-yl]-phenyl-methanone 10b

Yellow solid was obtained according to the previous general preparation. Yield (1.63 g, 83%); mp (173–175) °C; IR (KBr) ν_max_: 3249 (NH), 1685 (C=O), (1613–1600) cm^−1^ (C=C); ^1^H NMR (400 MHz, DMSO-d6) δ 2.17 (s, 3H, CH_3_ of thiophene), 3.49 (S, 3H, NCH_3_), 5.32 (d, 1H, H-4 of pyrimidine, *J* = 5.7 Hz), 6.07 (d, 1H, H-5 of pyrimidine, *J* = 7.4 Hz), 6.71 (d, 1H, H-3 of pyrrole, *J* = 6.6 Hz), 7.01 (dd, 1H, H-4 of pyrrole), 7.19 (d, 1H, H-5 of pyrrole, *J* = 6.8 Hz), (7.34–8.18) (m, 7H, of thiophene and phenyl), 11.34 ppm (br, 1H, NH, D_2_O exchangeable). Anal. Calcd for C_21_H_19_N_3_OS_2_ (393.53**):** C, 64.04; H, 4.83; N, 11.67. Found: C, 63.95; H, 4.78; N, 11.68.

##### [6-(5-Chloro-thiophen-2-yl)-4-(1-methyl-1H-pyrrol-2-yl)-2-thioxo-3,6-dihydro-2H-pyrimidin-1-yl]-phenyl-methanone 10c

According to the previous general procedure a yellow powder was obtained. Yield (1.82 g, 88%); mp (178–180) °C; IR (KBr) ν_max_: 3263 (NH), 1697 (C=O), (1615–1602) cm^−1^ (C=C); ^1^H NMR (400 MHz, DMSO-d6) δ 3.61 (S, 3H, NCH_3_), 5.73 (d, 1H, H-4 of pyrimidine, *J* = 7.9 Hz), 6.23 (d, 1H, H-5 of pyrimidine, *J* = 6.1 Hz), (7.34–8.18) (m, 10H, of aryl protons), 11.42 ppm (br, 1H, NH, D_2_O exchangeable). (*m/z*): 413 (M^+^, 413, M + 2^+^, 415) (3:1) ratio. Anal. Calcd for C_20_H_16_N_3_OS_2_Cl (413.95): C, 57.97; H, 3.86; N, 10.15. Found: C, 57.89; H, 3.79; N, 10.12.

#### General method for synthesis of 7-(1-Methyl-1H-pyrrol-2-yl)-5-(5-substituted-thiophen-2-yl)-3-phenyl-5H-[1,2,4]thiadiazolo[4,5-a]pyrimidine 11a-c

A solution of compounds ***10a-c*** (3 mmol), 10% sodium hypochlorite (10 mL), 10 mL NH_4_OH and 10% of NaOH (10 mL) was heated under reflux for 3 h. The reaction mixture was left to cool down and poured into ice water, filtered and recrystallized from ethanol to obtain colored compounds ***11a-c*.**

##### 7-(1-Methyl-1H-pyrrol-2-yl)-3-phenyl-5-thiophen-2-yl-5H-[1,2,4]thiadiazolo[4,5-a]pyrimidine 11a

Accordi ng to the previous general preparation method yellow powder was obtained. Yield (0.73 g, 64%); mp (124–126) °C; IR (KBr) ν_max_: 1624, 1631 (2C=N), (1608–1599) cm^−1^ (C=C); ^1^H NMR (400 MHz, DMSO-d6) δ 3.58 (S, 3H, NCH_3_), 5.64 (d, 1H, H-4 of pyrimidine, *J* = 5.9 Hz), 6.28 (d, 1H, H-5 of pyrimidine, *J* = 7.4 Hz), (6.57–8.26) ppm (m, 12H, of pyrrole thiophene and phenyl). ^13^C NMR (100 MHz, DMSO-d6) δ 53.76 (NCH_3_), 65.13 (C-4 of pyrimidine), 118.34, 120.15, 124.66, 127.13, 130.87, 133.23, 137.22 139.88, 141.22 (17C, C-aryl), 150.11, 153.34 ppm (2C=N). Anal. Calcd for C_20_H_16_N_4_S_2_ (376.50): C, 63.75; H, 4.25; N, 14.87. Found: C, 63.69; H, 4.19; N, 14.82.

##### 7-(1-Methyl-1H-pyrrol-2-yl)-5-(5-methyl-thiophen-2-yl)-3-phenyl-5H-[1,2,4]thiadiazolo[4,5-a]pyramid- ine 11b

A yellow powder of ***10b*** was obtained according to the aforementioned preparation method.Yield (0.70 g, 60%); mp (129–131) °C; IR (KBr) ν_max_: 1619, 1627 (2C=N), (1610–1602) cm^−1^ (C=C); ^1^H NMR (400 MHz, DMSO-d6) δ 2.31 (s, 3H, CH_3_ of thiophene), 3.41 (S, 3H, NCH_3_), 5.42 (d, 1H, H-4 of pyrimidine, *J* = 8.1 Hz), 6.19 (d, 1H, H-5 of pyrimidine, *J* = 6.2 Hz), 6.43 (d, 1H, H-3 of pyrrole, *J* = 6.8 Hz), 7.06 (dd, 1H, H-4 of pyrrole), 7.17 (d, 1H, H-5 of pyrrole, *J* = 6.8 Hz), (7.31–8.17) ppm (m, 7H, of thiophene and phenyl). Anal. Calcd for C_21_H_18_N_4_S_2_ (390.53**):** C, 64.53; H, 4.61; N, 14.34. Found: C, 64.46; H, 4.53; N, 14.54.

##### 5-(5-Chloro-thiophen-2-yl)-7-(1-methyl-1H-pyrrol-2-yl)-3-phenyl-5H-[1,2,4]thiadi- azolo[4,5-a]pyrimidine 11c

According to the previous general procedure a yellow powder was obtained.Yield (0.84 g, 68%); mp (137–139) °C; IR (KBr) ν_max_: 1615, 1623 (2C=N), (1612–1593) cm^−1^ (C=C); ^1^H NMR (400 MHz, DMSO-d6) δ 3.53 (S, 3H, NCH_3_), 5.64 (d, 1H, H-4 of pyrimidine, *J* = 5.9 Hz), 6.31 (d, 1H, H-5 of pyrimidine), 6.52 (d, 1H, H-3 of pyrrole, *J* = 6.4 Hz), 7.11 (dd, 1H, H-4 of pyrrole), 7.25 (d, 1H, H-5 of pyrrole, *J* = 6.3 Hz), (7.45–8.28) ppm (m, 7H, of thiophene and phenyl). (*m/z*): 410 (M^+^, 410, M + 2^+^, 412) (3:1) ratio. Anal. Calcd for C_20_H_15_N_4_S_2_Cl (410.94): C, 58.39; H, 3.65; N, 13.63. Found: C, 58.25; H, 3.59; N, 13.57.

#### General method for synthesis of [4-(1-Methyl-1H-pyrrol-2-yl)-6-(5-substituted-thiophen-2-yl)-1,6-dihydro-pyrimidin-2-yl]-hydrazine 12a-c

A mixture of pyrimidine-2-thione ***4a-c*** (5 mmol) and 10 mL hydrazine hydrate in 30 mL ethanol was refluxed for 6 h. The reaction mixture was allowed to cool and poured onto ice water. After filtration the crystallization took place from ethanol to obtain the corresponding hydrazine derivatives ***12a-c*.**

##### [4-(1-Methyl-1H-pyrrol-2-yl)-6-thiophen-2-yl-1,6-dihydro-pyrimidin-2-yl]-hydrazine 12a

After recrystallization of the product from ethanol according to the previous general preparation method, pale yellow powder was obtained. Yield (1.08 g, 79%); mp (167–169) °C; IR (KBr) ν_max_: (33,025–3177) (2NH, NH_2_), 1617 (C=N); ^1^H NMR (400 MHz, DMSO-d6) δ 3.32 (S, 3H, NCH_3_), 4.62 ppm (s, br, 2H, NH_2_, D_2_O exchangeable), 4.83 (d, 1H, H-4 of pyrimidine, *J* = 6.3 Hz), 6.21 (d, 1H, H-5 of pyrimidine, *J* = 7.8 Hz), 6.67 (d, 1H, H-3 of pyrrole, *J* = 6.3 Hz), 7.17 (dd, 1H, H-4 of pyrrole), 7.27 (d, 1H, H-5 of pyrrole, *J* = 6.9 Hz), 7.37 (d, 1H, H-3 of thiophene, *J* = 5.2 Hz), 7.73 (dd, 1H, H-4 of thiophene), 8.06 (d, 1H, H-5 of thiophene, *J* = 6.5 Hz), 9.11 (s, br, H, 1NH of pyrimidine, D_2_O exchangeable), 10.39 ppm (br, 1H, NH of hydrazine, D_2_O exchangeable). Anal. Calcd for C_13_H_15_N_5_S (273.36): C, 57.07; H, 5.48; N, 25.61. Found: C, 56.98; H, 5.43; N, 25.57.

##### [4-(1-Methyl-1H-pyrrol-2-yl)-6-(5-methyl-thiophen-2-yl)-1,6-dihydro-pyrimidin-2-yl]-hydrazine 12b

According to the previous general preparation method, yellow powder was obtained.. Yield (1.07 g, 75%); mp (174–176) °C; IR (KBr) ν_max_: (3367–3181) (2NH, NH_2_), 1632 (C=N), (1619–1604) cm^−1^ (C=C); ^1^H NMR (400 MHz, DMSO-d6) δ 2.09 (s, 3H, CH_3_ of thiophene), 3.49 (S, 3H, NCH_3_), 4.46 ppm (s, br, 2H, NH_2_, D_2_O exchangeable), 4.90 (d, 1H, H-4 of pyrimidine, *J* = 5.8 Hz), 6.35 (d, 1H, H-5 of pyrimidine, *J* = 7.4 Hz), 6.73 (d, 1H, H-3 of pyrrole, *J* = 6.5 Hz), 7.23 (dd, 1H, H-4 of pyrrole), 7.43 (d, 1H, H-5 of pyrrole, *J* = 6.7 Hz), 7.56 (d, 1H, H-3 of thiophene, *J* = 5.8 Hz), 7.90 (d, 1H, H-4 of thiophene*, J* = 6.7 Hz), 8.79 (s, br, H, 1NH of pyrimidine, D_2_O exchangeable), 9.85 ppm (br, 1H, NH of hydrazine, D_2_O exchangeable). Anal. Calcd for C_14_H_17_N_5_S (287.39): C, 58.46; H, 5.92; N, 24.36. Found: C, 58.42; H, 5.86; N, 24.29.

##### [6-(5-Chloro-thiophen-2-yl)-4-(1-methyl-1H-pyrrol-2-yl)-1,6-dihydro-pyrimidin-2-yl]-hydrazine 12c

According to the previous general preparation method, yellow powder was obtained.. Yield (1.23 g, 80%); mp (182–184) °C; IR (KBr) ν_max_: (3376–3173) (2NH, NH_2_), 1635 (C=N), (1623–1608) cm^−1^ (C=C); ^1^H NMR (400 MHz, DMSO-d6) δ 3.64 (S, 3H, NCH_3_), 4.65 ppm (s, br, 2H, NH_2_, D_2_O exchangeable), 5.08 (d, 1H, H-4 of pyrimidine, *J* = 6.1 Hz), 6.57 (d, 1H, H-5 of pyrimidine), 6.61 (d, 1H, H-3 of pyrrole, *J* = 6.3 Hz), 7.28 (dd, 1H, H-4 of pyrrole), 7.38 (d, 1H, H-5 of pyrrole, *J* = 7.0 Hz), 7.82 (d, 1H, H-3 of thiophene, *J* = 6.6 Hz), 8.18 (d, 1H, H-4 of thiophene*, J* = 7.2 Hz), 9.32 (s, br, H, 1NH of pyrimidine, D_2_O exchangeable), 10.49 ppm (br, 1H, NH of hydrazine, D_2_O exchangeable). Anal. Calcd for C_13_H_14_N_5_SCl (307.80): C, 50.68; H, 4.55; N, 22.74. Found: C, 50.63; H, 4.49; N, 22.68.

#### General method for synthesis of 5-Methyl-2-[4-(1-methyl-1H-pyrrol-2-yl)-6-(5-methyl-thiophen-2-yl)-1,6-dihydro-pyrimidin-2-yl]-2,4-dihydro-pyrazol-3-one. 13a-c

A solution of compound ***12a-c*** (4 mmol) and 10 mL ethyl acetoacetate in 20 mL acetic acid was heated under reflux for 5 h. The reaction mixture was allowed to cool and poured onto ice water. After filtration, the obtained product was dried and recrystallized from ethanol to obtain the corresponding pyrazol-3-one derivatives ***13a-c*.**

##### 5-Methyl-2-[4-(1-methyl-1H-pyrrol-2-yl)-6-thiophen-2-yl-1,6-dihydro-pyrimidin-2-yl]-2,4-dihydro-pyrazol-3-one 13a

According to the previous general preparation method, dark yellow crystals were obtained. Yield (0.99 g, 73%); mp (196–198) °C; IR (KBr) ν_max_: (3176) (NH), 1692 (C=O), 1637, 1625 (2 C=N); ^1^H NMR (400 MHz, DMSO-d6) δ 1.89 (s, 3H, CH_3_ of pyrazol), 2.89 (s, 2H, CH_2_ of pyrazol), 3.63 (S, 3H, NCH_3_), 5.94 (d, 1H, H-4 of pyrimidine, *J* = 5.4 Hz), 6.44 (d, 1H, H-5 of pyrimidine, *J* = 7.9 Hz), 6.73 (d, 1H, H-3 of pyrrole, *J* = 6.5 Hz), 7.31 (dd, 1H, H-4 of pyrrole), 7.51 (d, 1H, H-5 of pyrrole, *J* = 6.4 Hz), 7.48 (d, 1H, H-3 of thiophene, *J* = 5.3 Hz), 7.82 (dd, 1H, H-4 of thiophene), 8.17 (d, 1H, H-5 of thiophene, *J* = 6.8 Hz), 9.38 ppm (s, br, H, 1NH of pyrimidine, D_2_O exchangeable). ^13^C NMR (100 MHz, DMSO-d6) δ 25.54 (CH_3_), 49.34 (NCH_3_), 69.75 (C-4 of pyrimidine), 119.19, 121.33, 125.55, 127.32, 130.78, 135.59, 138.84, 145.77 (11 C of aryl C), 158.73, 160.22 (2C=N), 177.04 ppm (C=O). Anal. Calcd for C_17_H_17_N_5_OS (339.42): C, 60.10; H, 5.01; N, 20.62. Found: C, 59.97; H, 4.97; N, 20.60.

##### 5-Methyl-2-[4-(1-methyl-1H-pyrrol-2-yl)-6-(5-methyl-thiophen-2-yl)-1,6-dihydro-pyrimidin-2-yl]-2,4-dihydro-pyrazol-3-one 13b

Dark yellow crystals were obtained according to the previous general preparation method. Yield (0.97 g, 69%); mp (189–191) °C; IR (KBr) ν_max_: (3178) (NH), 1683 (C=O), 1642, 1629 (2 C=N), (1615–1603) cm^−1^ (C=C); ^1^H NMR (400 MHz, DMSO-d6) δ 1.75 (s, 3H, CH_3_ of pyrazol), 2.13 (s, 3H, CH_3_ of thiophene), 2.73 (s, 2H, CH_2_ of pyrazol), 3.43 (S, 3H, NCH_3_), 5.46 (d, 1H, H-4 of pyrimidine, *J* = 6.7 Hz), 6.24 (d, 1H, H-5 of pyrimidine, *J* = 7.3 Hz), 6.81 (d, 1H, H-3 of pyrrole, *J* = 7.0 Hz), 7.29 (dd, 1H, H-4 of pyrrole), 7.42 (d, 1H, H-5 of pyrrole, *J* = 6.8 Hz), 7.57 (d, 1H, H-3 of thiophene, *J* = 6.8 Hz), 7.98 (d, 1H, H-4 of thiophene), 10.51 ppm (s, br, H, 1NH of pyrimidine, D_2_O exchangeable). Anal. Calcd for C_18_H_19_N_5_OS (353.44): C, 61.11; H, 5.38; N, 19.81. Found: C, 61.04; H, 5.35; N, 19.74.

##### 2-[6-(5-Chloro-thiophen-2-yl)-4-(1-methyl-1H-pyrrol-2-yl)-1,6-dihydro-pyrimidin-2-yl]-5-methyl-2,4-dihydro-pyrazol-3-one 13c

Brown crystals were obtained according to the previous general preparation method. Yield (1.12 g, 75%); mp (205–207) °C; IR (KBr) ν_max_: (3208) (NH), 1688 (C=O), 1637, 1625 (2 C=N), (1617–1601) cm^−1^ (C=C); ^1^H NMR (400 MHz, DMSO-d6) δ 1.82 (s, 3H, CH_3_ of pyrazol), 3.09 (s, 2H, CH_2_ of pyrazol), 3.51 (S, 3H, NCH_3_), 5.29 (d, 1H, H-4 of pyrimidine, *J* = 6.1 Hz), 6.11 (d, 1H, H-5 of pyrimidine, *J* = 6.9 Hz), 6.88 (d, 1H, H-3 of pyrrole, *J* = 7.5 Hz), 7.24 (dd, 1H, H-4 of pyrrole), 7.52 (d, 1H, H-5 of pyrrole, *J* = 6.1 Hz), 7.71 (d, 1H, H-3 of thiophene, *J* = 6.3 Hz), 8.23 (d, 1H, H-4 of thiophene, *J* = 7.2 Hz), 10.32 ppm (s, br, H, 1NH of pyrimidine, D_2_O exchangeable). (*m/z*): 373 (M^+^, 373, M + 2^+^, 375) (3:1) ratio. Anal. Calcd for C_17_H_16_N_5_OSCl (373.86): C, 54.57; H, 4.28; N, 18.72. Found: C, 54.48; H, 4.22; N, 18.66.

#### General method for synthesis of 7-(1-Methyl-1H-pyrrol-2-yl)-5-(5-substitured-thiophen-2-yl)-1,5-dihydro-[1,2,4]triazolo[4,3-a]pyrimidine 14a-c

A mixture of compound ***13a-c*** (4 mmol) and 20 mL formic acid was heated under reflux for 7 h. The reaction mixture was allowed to cool and poured onto ice water. After filtration, the obtained product was dried and recrystallized from ethanol to afford the corresponding [1, 2, 4]triazolo[4,3-a]pyrimidine ***14a-c***.

##### 7-(1-Methyl-1H-pyrrol-2-yl)-5-thiophen-2-yl-1,5-dihydro-[1,2,4]triazolo[4,3-a]pyrimidine 14a

According to the previous general preparation method, brown crystals were obtained. Yield (0.74 g, 65%); mp (178–180) °C; IR (KBr) ν_max_: (3275) (NH), 1643, 1632 (2 C=N), (1611–1594) cm^−1^ (C=C); ^1^H NMR (400 MHz, DMSO-d6) δ 3.37 (S, 3H, NCH_3_), 5.38 (d, 1H, H-4 of pyrimidine, *J* = 6.0 Hz), 6.23 (d, 1H, H-5 of pyrimidine, *J* = 7.3 Hz), (6.81–7.53) (m, 3H, of pyrrole), (7.89–8.11) (m, 3H, of thiophene), 8.42 ppm (s, H-3 of triazole), 12.83 ppm (s, br, H, 1NH of trizole, D_2_O exchangeable). Anal. Calcd for C_14_H_13_N_5_S (283.35): C, 59.29; H, 4.59; N, 24.70. Found: C, 59.16; H, 4.54; N, 24.62.

##### 7-(1-Methyl-1H-pyrrol-2-yl)-5-(5-methyl-thiophen-2-yl)-1,5-dihydro-[1,2,4]triazolo[4,3-a]pyrimidine. 14b

Brown crystals were obtained according to the previous general preparation method. Yield (0.81 g, 68%); mp (174–176) °C; IR (KBr) ν_max_: (3251) (NH), 1637, 1626 (2 C=N), (1613–1598) cm^−1^ (C=C); ^1^H NMR (400 MHz, DMSO-d6) δ 1.99 (s, 3H, CH_3_ of thiophene), 3.42 (S, 3H, NCH_3_), 5.19 (d, 1H, H-4 of pyrimidine, *J* = 6.7 Hz), 6.09 (d, 1H, H-5 of pyrimidine, *J* = 7.7 Hz), 6.57 (d, 1H, H-3 of pyrrole, *J* = 6.3 Hz), 7.08 (dd, 1H, H-4 of pyrrole), 7.34 (d, 1H, H-5 of pyrrole, *J* = 5.7 Hz), 7.72 (d, 1H, H-3 of thiophene, *J* = 6.1 Hz), 7.99 (d, 1H, H-4 of thiophene,, *J* = 6.3 Hz), 8.25 ppm (s, H-3 of triazole), 12.09 ppm (s, br, H, 1NH of trizole, D_2_O exchangeable). Anal. Calcd for C_15_H_15_N_5_S (297.38**):** C, 60.53; H, 5.04; N, 23.54. Found: C, 60.51; H, 5.04; N, 23.57.

##### 5-(5-Chloro-thiophen-2-yl)-7-(1-methyl-1H-pyrrol-2-yl)-1,5-dihydro-[1,2,4]triazolo[4,3-a]pyrimidine. 14c

Dark brown crystals were obtained according to the previous general preparation method. Yield (0.92 g, 72%); mp (182–184) °C; IR (KBr) ν_max_: (3272) (NH), 1635, 1621 (2 C=N), (1608–1591) cm^−1^ (C=C); ^1^H NMR (400 MHz, DMSO-d6) δ 3.27 (S, 3H, NCH_3_), 5.08 (d, 1H, H-4 of pyrimidine, *J* = 7.8 Hz), 6.11 (d, 1H, H-5 of pyrimidine, *J* = 7.9 Hz), 6.61 (d, 1H, H-3 of pyrrole, *J* = 6.8 Hz), 7.01 (dd, 1H, H-4 of pyrrole), 7.27 (d, 1H, H-5 of pyrrole, *J* = 6.5 Hz), 7.61 (d, 1H, H-3 of thiophene, *J* = 7.3 Hz), 7.74 (d, 1H, H-4 of thiophene,, *J* = 6.8 Hz), 8.36 ppm (s, H-3 of triazole), 12.56 ppm (s, br, H, 1NH of trizole, D_2_O exchangeable). Anal. Calcd for C_14_H_12_N_5_SCl (317.80): C, 52.86; H, 3.78; N, 22.03. Found: C, 52.72; H, 3.73; N, 21.96.

## Conclusion

New condensed and non-condensed heterocyclic compounds based on pyrimidine-2-thiones **4a-c** were synthesized. The first synthetic path way took place through *S*-alkylation of pyrimidine-2-thiones **4a-c** followed by reaction with ammonia to produce the corresponding thiazolo[3,2-a]pyrimidin-3-ones **7a-c** which underwent condensation with benzaldehyde followed by heating under reflux with hydroxylamine afforded the corresponding isoxazolo [5′,4′:4,5]thiazolo[3,2-a]pyrimidinse **9a-c**. The second path way of this work was the heatng of the key synthons **4a-c** with benzoylcholride followed by reaction with sodium hypochlorite, ammonia and sodium hydroxide to produce [1, 2, 4]thiadiazolo[4,5-a]pyrimidine **11a-c**. A final route of this work was the hydrazinolysis of **4a-c** followed by the cyclocondensation with ethyl acetoacetate or formic acid to produce pyrazol-3-ones **13a-c** or [1, 2, 4]triazolo[4,3-a]pyrimidine **14a-c**, respectively. All newly synthesized heterocyclic structures were confirmed using various tools including, elemental analysis, IR, ^1^H-NMR, ^13^C-NMR and mass spectra. Screening of the selected compounds **4a, 6a, 7a, 9a, 10a, 13a** and **14a** against colon carcinoma cells lines (HCT-116) and hepatocellular carcinoma cells lines (HepG-2) showed that the compound 2-thioxo-3,6-dihydro-2H-pyrimidin-1-yl]-phenyl-methanone **10a** was the most active among the group of selected compounds, meanwhile, compounds **4a, 6a** and **14a** exhibited considerable cytotoxic action, furthermore, compounds **7a, 9a and 13a** were showed weak cytotoxic action. These results encourage us to suggest that the compound **10a** be used in the formulation of antibiotics as a medication to improve the sensitivity of antibiotics that stimulate cancer therapy and cause apoptosis in both human colon carcinoma cancer and hepatocellular carcinoma.

## Supplementary Information

**Additional file 1:** a) Figures illustrating the IR spectra of compounds 4a, 6a,7a and 9a-13a.Figures illustrating the 1H NMR of compounds 4a, 7a-11a and 14a. b) Tables contain elemental analysis for all prepared compounds. c) Table containS melting points, yield % and IR spectral data of compounds 3a-c.

## Data Availability

The datasets used or analyzed during the current study are available from the corresponding author on reasonable request.
