# Supplementary material for: Synthesis, screening as potential antitumor of new poly heterocyclic compounds based on pyrimidine-2-thiones
Source: BMC Chem. 2022 Mar 21;16(1):16. doi: 10.1186/s13065-022-00810-4 (PMC8939104; doi:10.1186/s13065-022-00810-4)
Supplement: Supplementary file 1 — Additional file 1: a) Figures illustrating the IR spectra of compounds 4a, 6a,7a and 9a-13a.Figures illustrating the 1H NMR of compounds 4a, 7a-11a and 14a. b) Tables contain elemental analysis for all prepared compounds. c) Table containS melting points, yield % and IR spectral data of compounds 3a-c. [file 13065_2022_810_MOESM1_ESM.pdf]

<u>**I R and NMR Spectra**</u>

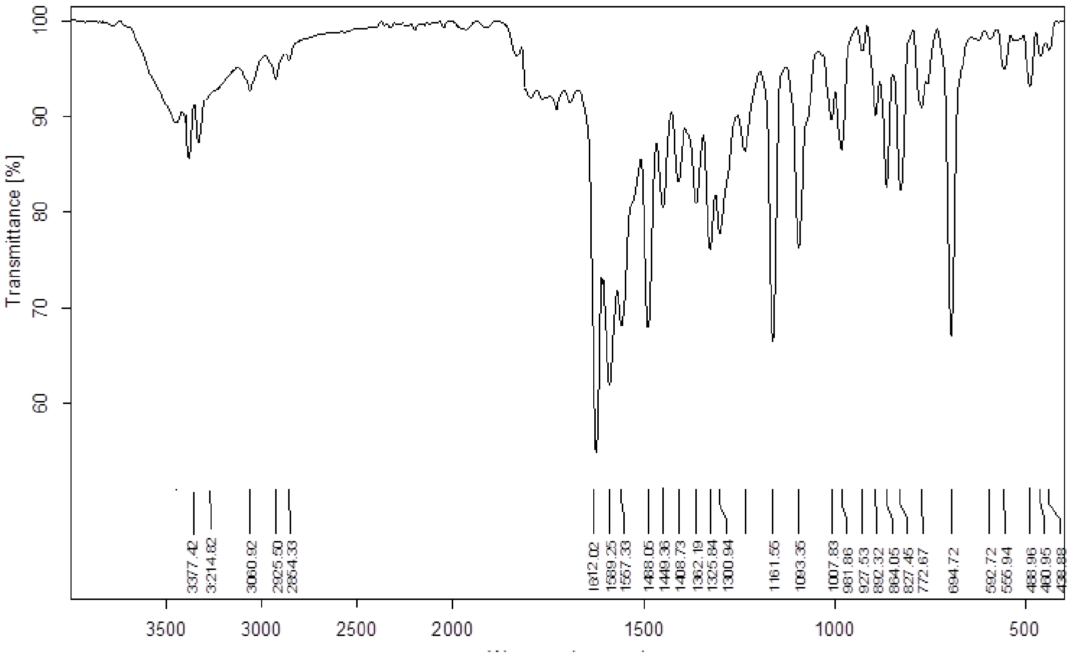

IR (KBr) of compound **4a**

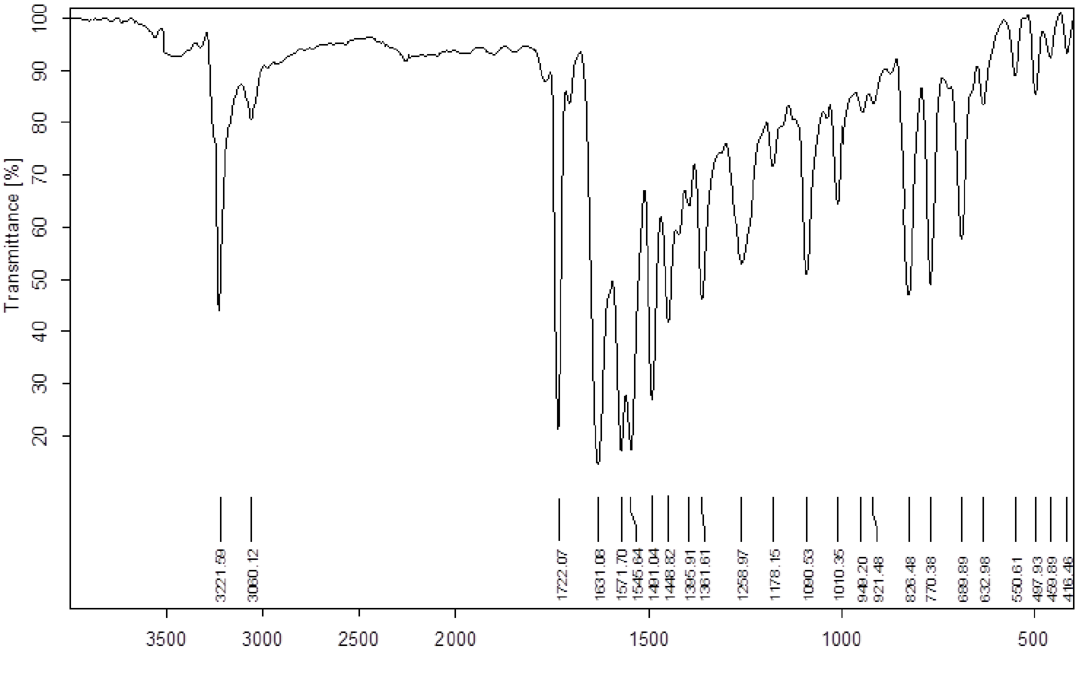

IR (KBr) of compound **6a**

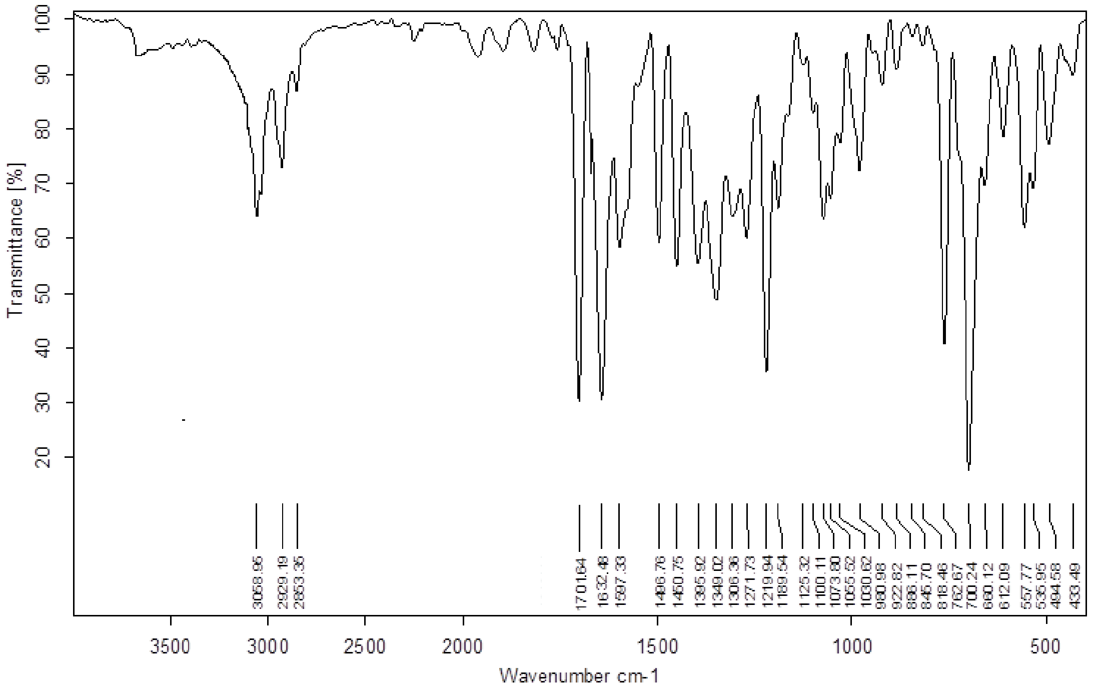

IR (KBr) of compound **7a**

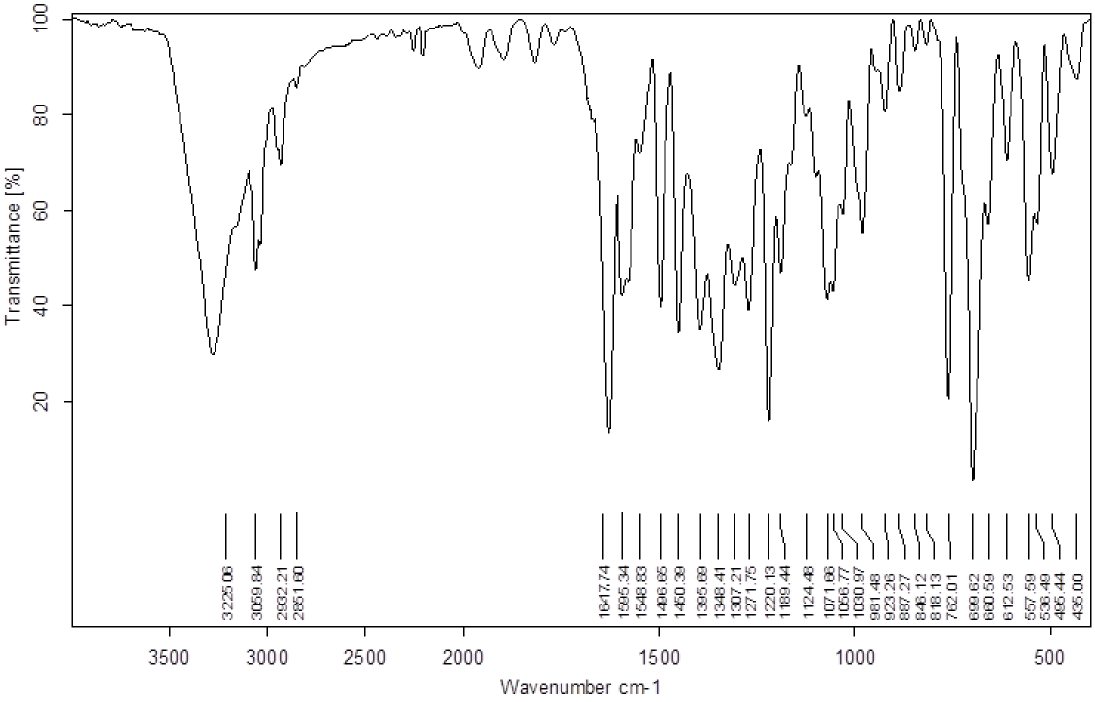

IR (KBr) of compound **9a**

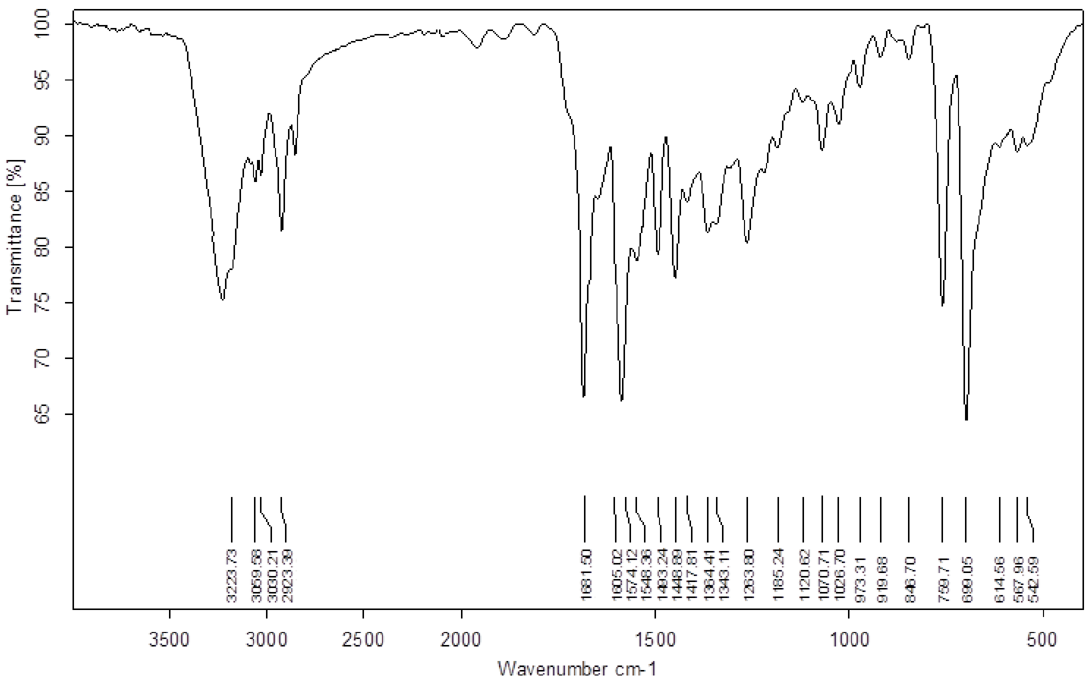

IR (KBr) of compound **10a**

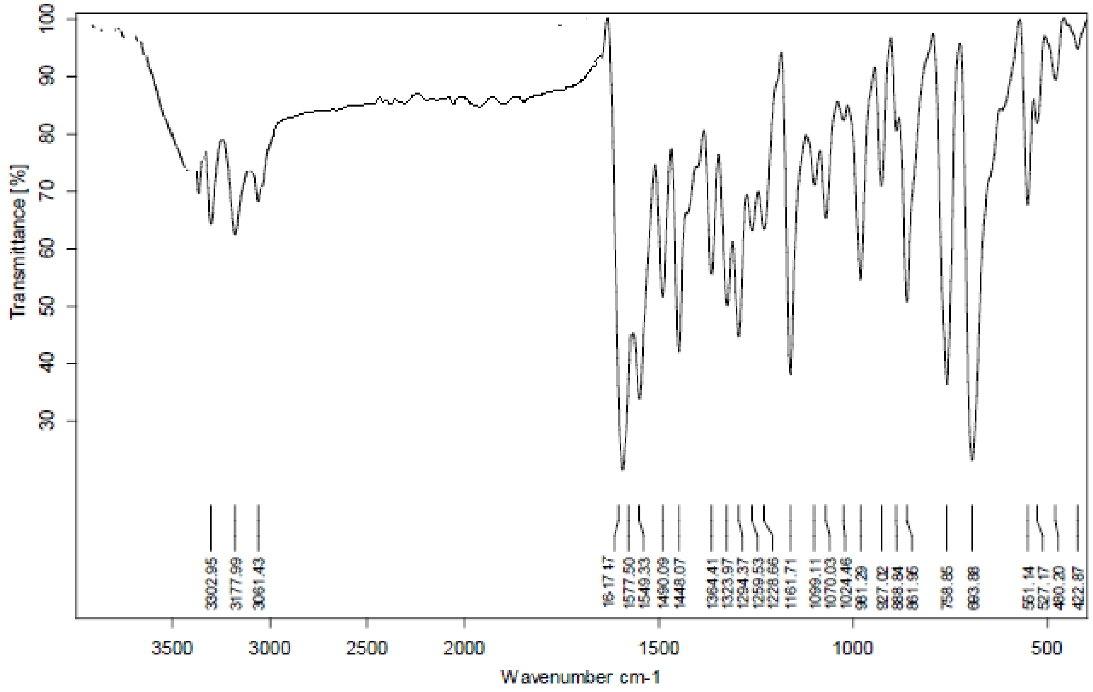

IR (KBr) of compound **12a**

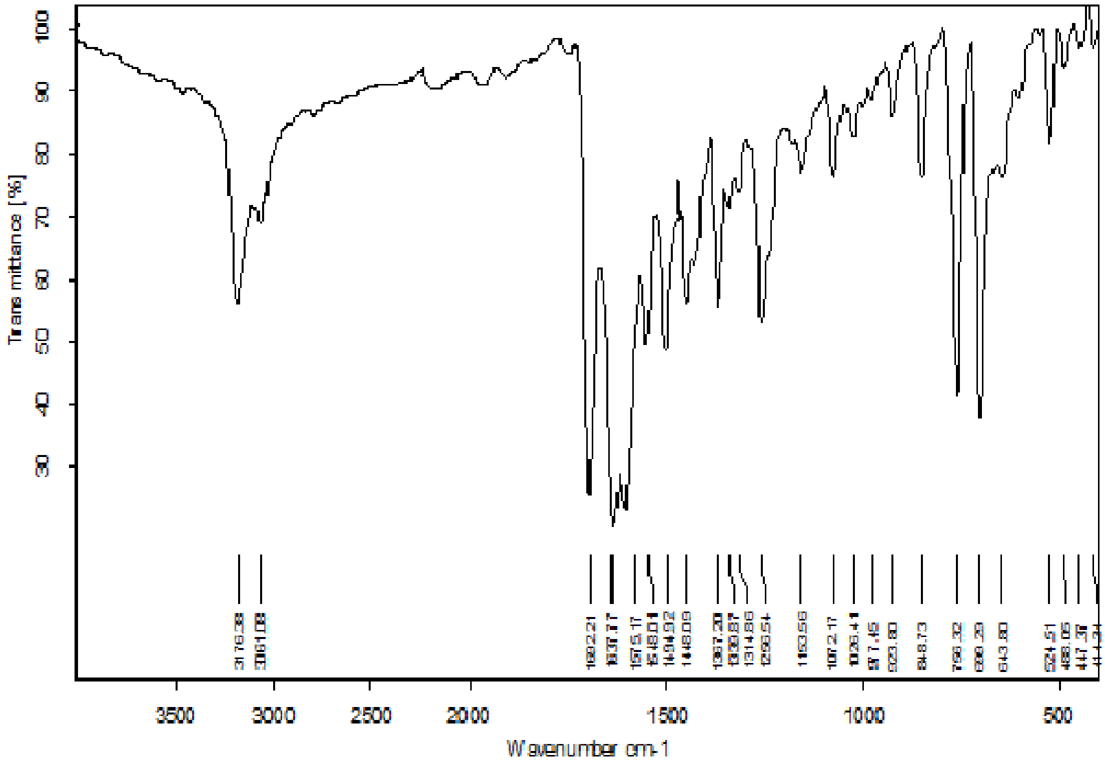

IR (KBr) of compound **13a**

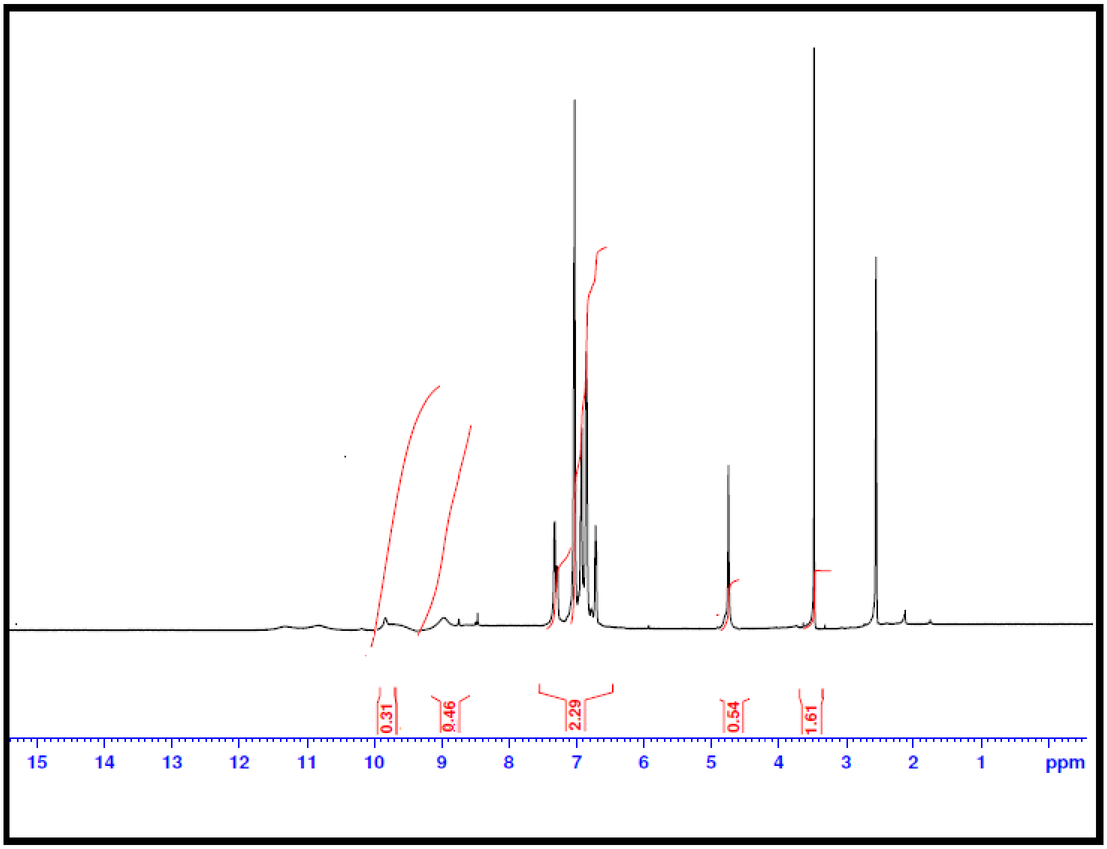

^{1}H-NMR of compound **4a** in DMSO-d6 at 400 MHz

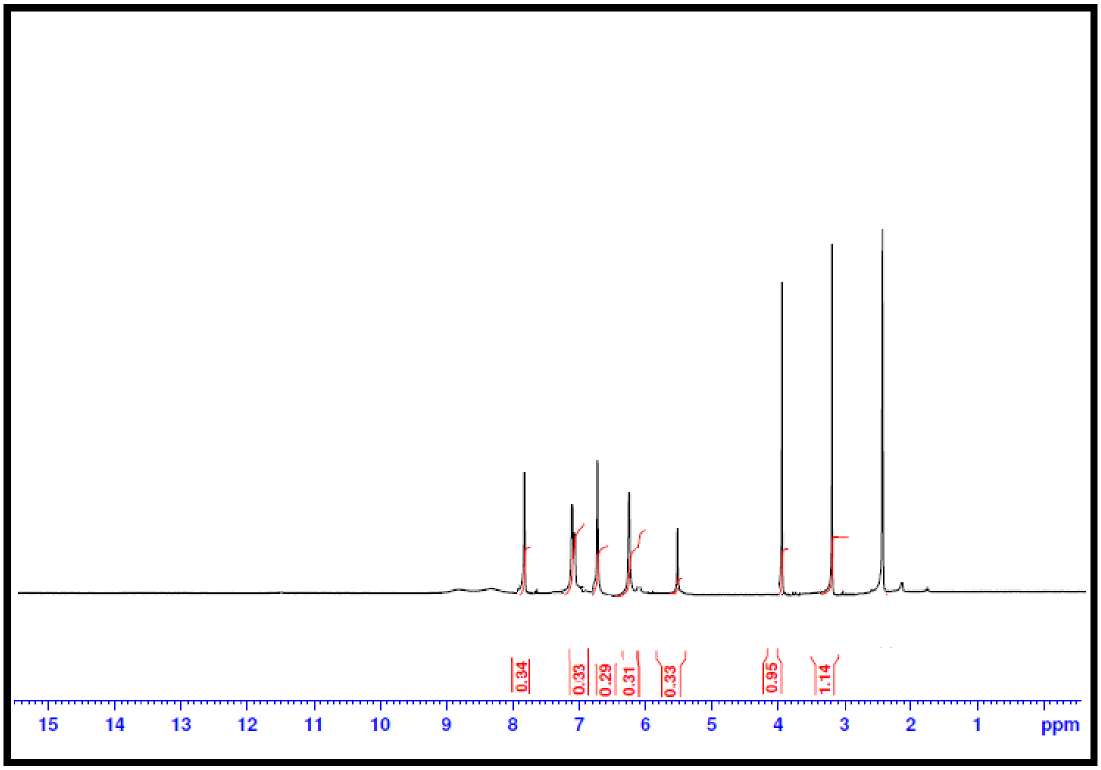

^{1}H-NMR of compound **7a** in DMSO-d6 at 400 MHz

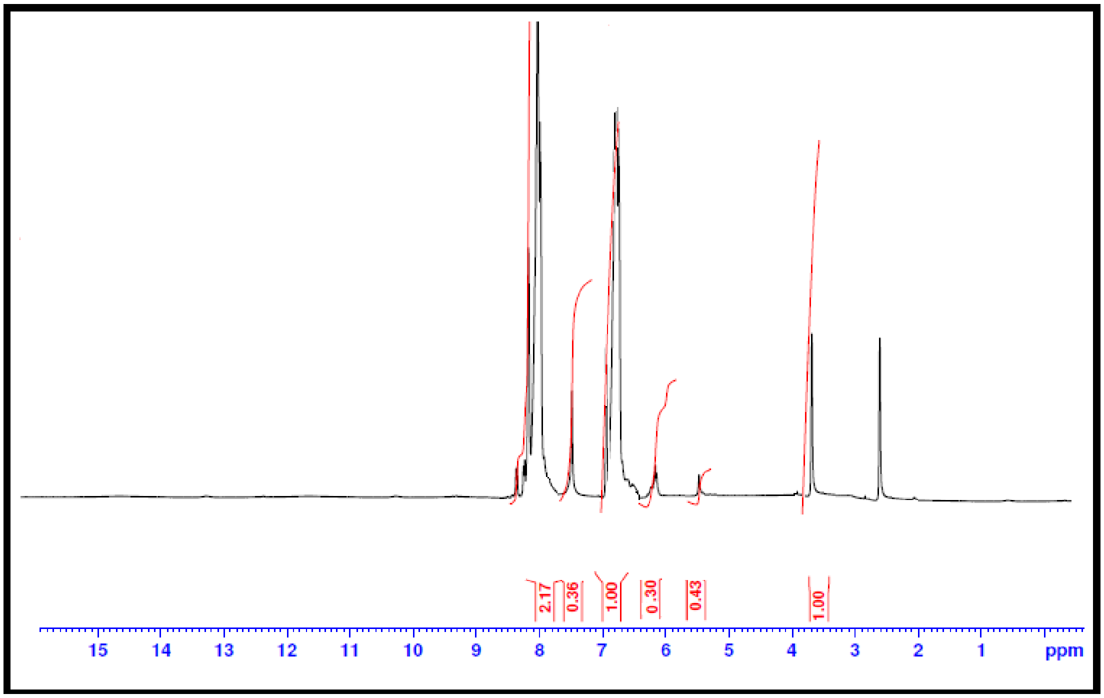

^{1}H-NMR of compound **8a** in DMSO-d6 at 400 MHz

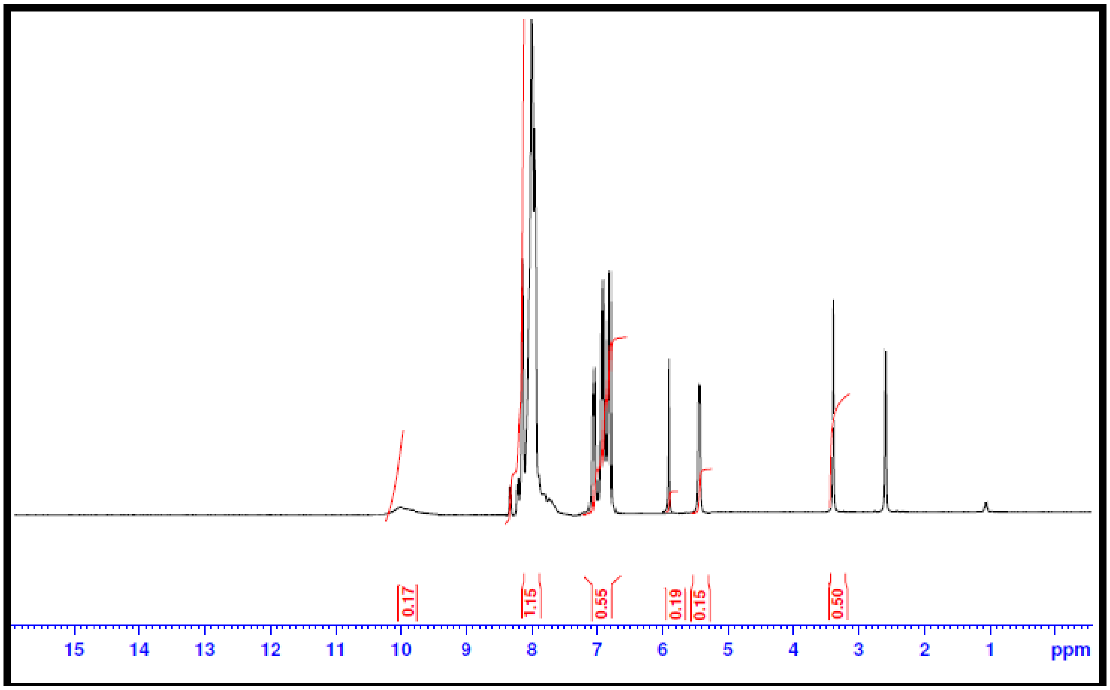

^{1}H-NMR of compound **9a** in DMSO-d6 at 400 MHz

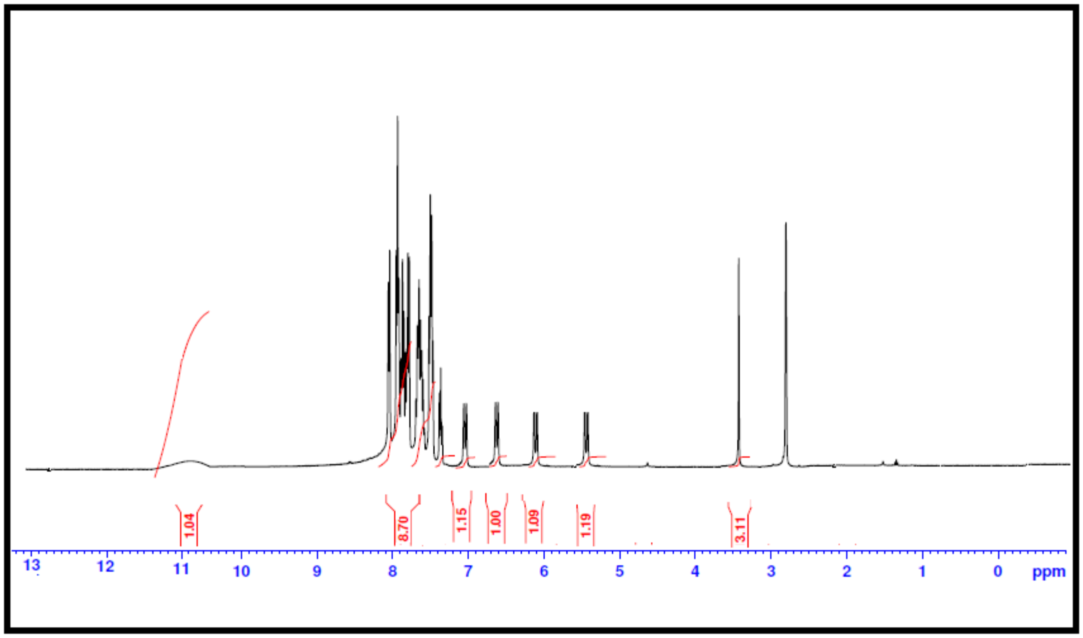

^{1}H-NMR of compound **10a** in DMSO-d6 at 400 MHz

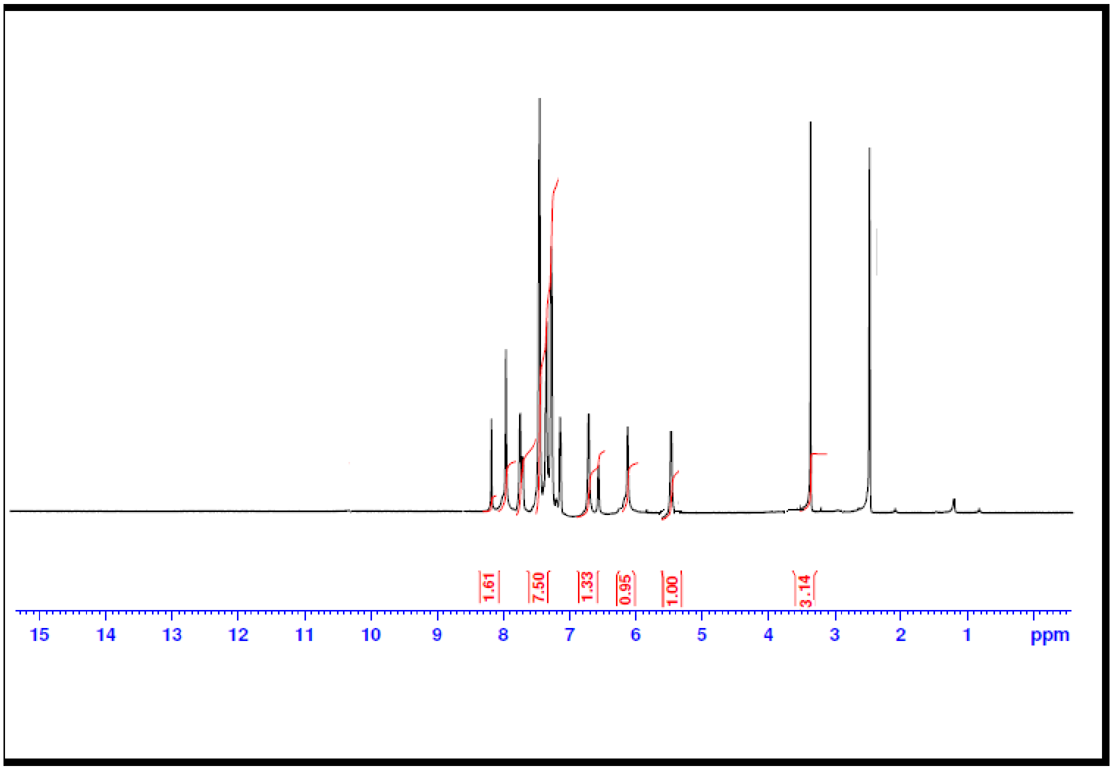

^{1}H-NMR of compound **11a** in DMSO-d6 at 400 MHz

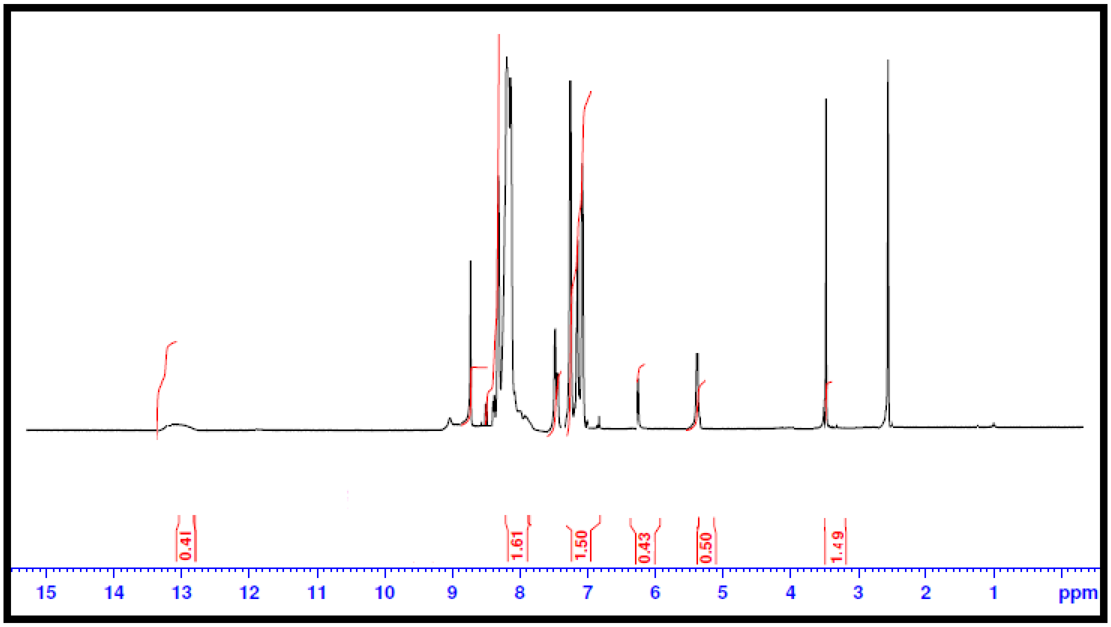

^{1}H-NMR of compound **14a** in DMSO-d6 at 400 MHz

Elemental analysis

Requester Data:
Name: Dr. El-sayed Abdelrehim .
<u>Sample data:</u>
Six Samples had been submitted for elemental analysis
<u>Analysis Report:</u>

Sample code	C %	H %	N %
4a	56.52	4.65	15.27
4b	58.11	5.14	14.43
4c	50.21	3.90	13.48
6a	56.45	5.28	11.57
6b	57.43	5.55	11.13
6c	51.47	4.49	10.57

INVESTIGATOR DIRECTOR

Requester Data:
Name: Dr. El-sayed. M Abdelrehim .
<u>Sample data:</u>
Nine Samples had been submitted for elemental analysis
<u>Analysis Report:</u>

Sample code	C %	H %	N %
7a	56.98	4.08	13.17
7b	58.17	4.49	12.67
7c	51.39	3.40	11.89
8a	65.33	4.18	10.39
8b	66.02	4.52	10.05
8c	60.19	3.58	9.53
9a	62.97	4.28	13.34
9b	63.85	4.57	12.86
9c	58.19	3.72	12.29

INVESTIGATOR DIRECTOR

Requester Data:
Name: Dr. El-sayed. M Abdelrehim .
<u>Sample data:</u>
Nine Samples had been submitted for elemental analysis
<u>Analysis Report:</u>

Sample code	C %	H %	N %
10a	63.18	4.42	10.97
10b	63.95	4.78	11.68
10c	57.89	3.79	10.12
11a	63.69	4.19	14.82
11b	64.46	4.53	14.54
11c	58.25	3.59	13.57
12a	56.98	5.43	25.57
12b	58.42	5.86	24.29
12c	50.63	4.49	22.68

INVESTIGATOR DIRECTOR

Requester Data:
Name: Dr. sayed. Abdelrehim .
Sample data:
Six Samples had been submitted for elemental analysis
Analysis Report:

Sample code	C %	H %	N %
13a	59.97	4.97	20.60
13b	61.04	5.35	19.74
13c	54.48	4.22	18.66
14a	59.16	4.54	24.62
14b	60.51	5.04	23.57
14c	52.72	3.73	21.96

INVESTIGATOR DIRECTOR

Table: Melting points, yield % and IR spectral data of compounds 3a-c

Entry	X	M.p ^{0}C	Yield %	IR (KBr) cm^{-1}	
				C=C	C=O
**3a**	H	143-145	82	(1605-1612)	1672
**3b**	CH_3	150-152	84	(1609-1618)	1681
**3c**	Cl	161-163	89	(1611-1620)	1679
